# Comparative quantification of local climate regulation by green and blue urban areas in cities across Europe

**DOI:** 10.1038/s41598-021-03140-y

**Published:** 2021-12-13

**Authors:** Romain Goldenberg, Zahra Kalantari, Georgia Destouni

**Affiliations:** Department of Physical Geography, Bolin Center for Climate Research and Navarino Environmental Observatory, Stockholm University, 10691 Stockholm, Sweden

**Keywords:** Ecosystem services, Urban ecology, Climate-change mitigation, Sustainability

## Abstract

Urban growth alters environmental conditions with major consequences for climate regulation and the exposure of population to heat. Nature-based solutions may be used to alleviate the increasing urban climate pressures, but the climate regulation services that these solutions can supply for and across different urban conditions remains understudied. We comparatively investigate the urban ecosystem service realization (considering the ecosystem service supply and demand spatial interactions) of local climate regulation by vegetated (green) and water-covered (blue) areas across 660 European cities. Results show relatively robust power-law relationships with city population density (average R^2^ of 0.34) of main indicators of ecosystem service realization. Country-wise fitting for city-average indicators strengthens these relationships, in particular for western European cities (average R^2^ of 0.66). Cross-city results also show strong power-law relationship of effectiveness in ecosystem service realization with socio-economic measures like Human Development Index and GPD per capita, in particular for the area fraction of city parts with high ecosystem service realization (R^2^ of 0.77). The quantified relationships are useful for comparative understanding of differences in ecosystem services realization between cities and city parts, and quantitative projection of possible change trends under different types of city growth so that relevant measures can be taken to counteract undesirable trends.

## Introduction

**Figure 1:**
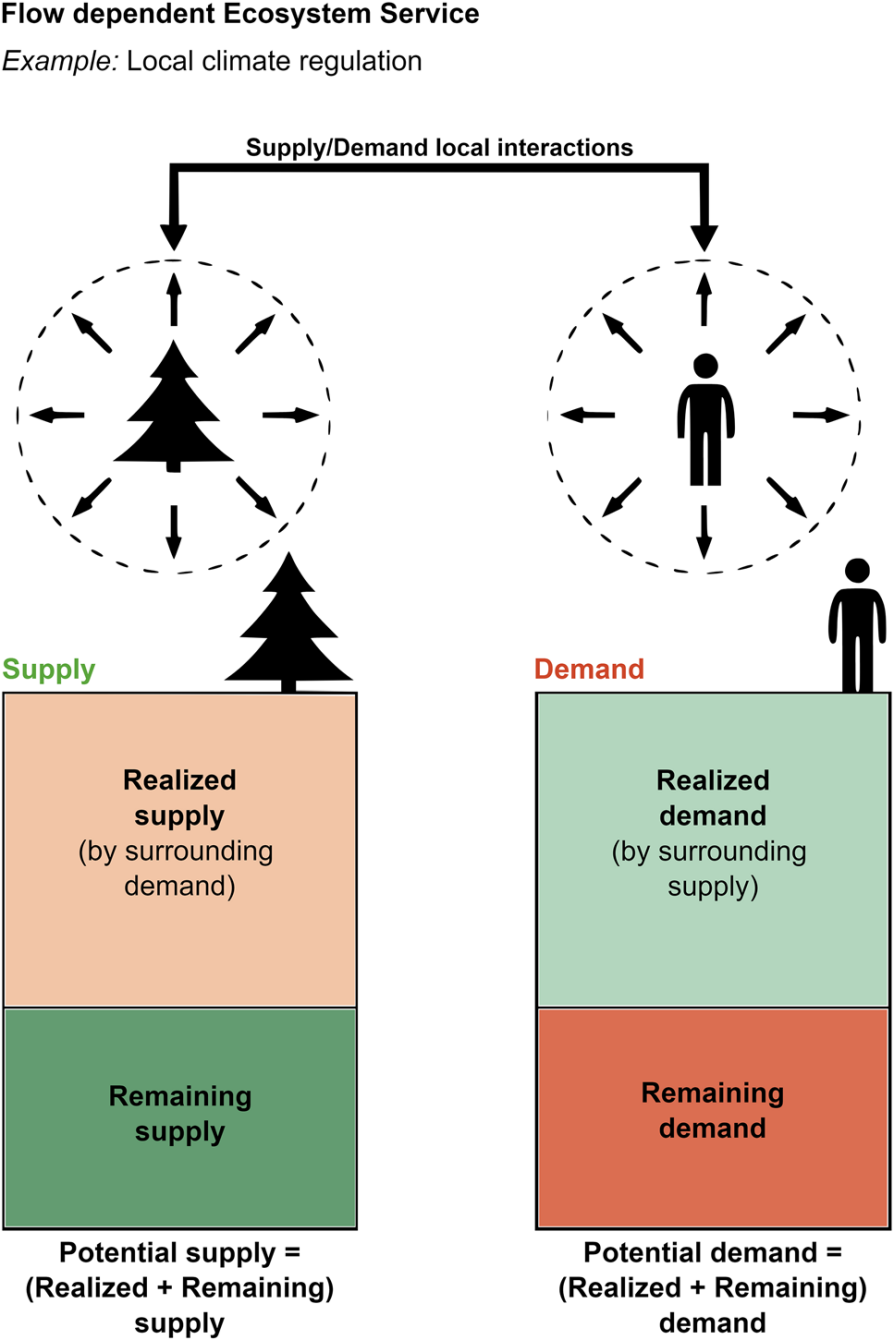
Spatial flow dependence of ecosystem services and studied city locations. Schematic of potential and realized supply and demand of flow-dependent ecosystem service (for explanation, see “Methods”).

Climate change and the urban heat island effect threaten the sustainability of rapidly growing urban settlements and urban population worldwide^1^. Such threats may be ameliorated by the ecosystem service of local climate regulation provided by green–blue urban areas (natural, restored, or (re)constructed ecosystems, such as forested land, wetlands, parks)^2–4^. The spatiotemporal relationships existing between natural ecosystems and human societies form the basis of the ecosystem service framework, used to represent such benefits from nature to human well-being^5,6^. Areas of ecosystem service provision (nature contribution of some supply) and ecosystem service use (human beneficiaries with some ecosystem service demand) in a landscape are then often connected by some form of carrier flow, which can be natural (air and water movement) or depend on human-made infrastructure (e.g., pipelines for water, road network and vehicles for human movement)^7,8^. Additionally, ecosystem service relevance is scale-dependent, e.g., with carbon sequestration being globally relevant, while recreational areas provide mostly local and regional benefits^9–11^. Over each scale of relevance, it is essential to distinguish the supply and demand sides of spatial ecosystem services relationships^2^, and the degree to which potential supply (left, Fig. 1) can actually reach and fulfill some actual demand (right, Fig. 1). This may be referred to as the degree of realization of ecosystem service supply and demand^12^. Conceptually, we define a potential as the hypothetical maximum capacity for a service (supply) or need (demand). In contrast, a realized service quantifies the actual ecosystem service, after consideration of proper spatial flow connections between natural ecosystems and humans. For example, for a city, only part of its total potential ecosystem service demand (Pd) may be actually fulfilled (referred to as the realized ecosystem service demand, Rd, right in Fig. 1) by only part (the realized supply part, Rs, left in Fig. 1) of the city’s total potential ecosystem service supply (Ps). Thus, Rd measures the part of the human demand (for the ecosystem service) actually fulfilled, while Rd quantify the part of the supply used to provide the ecosystem service. The Methods section describes and discusses in further detail this and other term definitions used in the analysis, the relationships between terms, and the calculation methods employed to quantify them.

**Figure 2:**
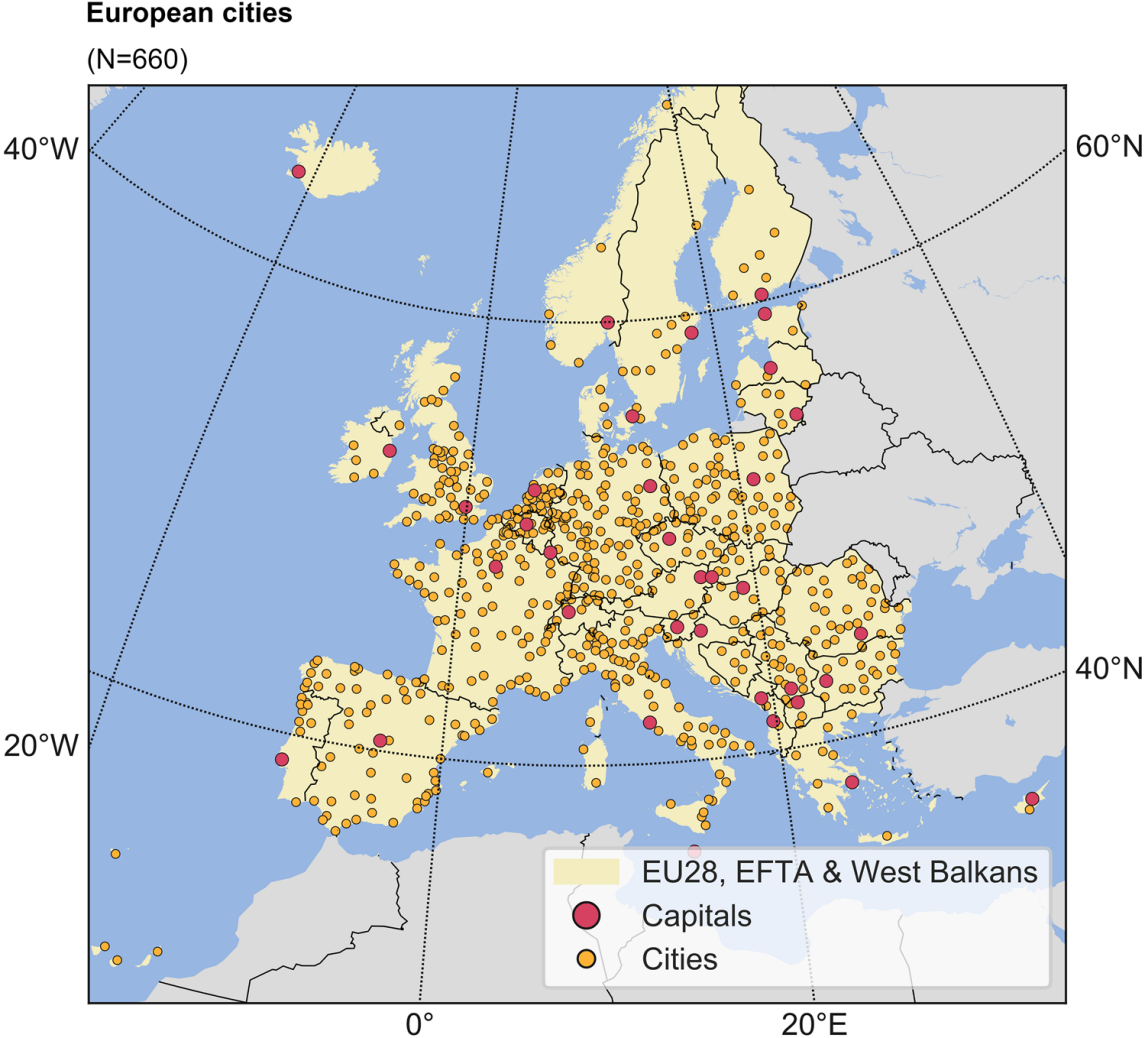
Studied city locations. Map of the European study region and locations of the cities studied. See Supplementary Table 1 for further city data.

In practice, implementing the concept of ecosystem services into urban landscape management and decision making is still problematic^5^, with one reason being the challenge to link spatially disaggregated areas of service provision with the human beneficiaries^13^. In addition, considerable ambiguity still remains, conceptually and in practice, regarding the distinction and quantification of potential and realized ecosystem services supply and demand^14^. For example, without consideration of the spatial relationship between supply and demand (implicitly or explicitly), it becomes difficult to determine or quantify, in practice, if an actual ecosystem service exists. To contribute to its resolution, we here investigate the degree of supply and demand realization for the urban ecosystem service of local climate regulation using comparative quantitative indicators in and across 660 cities of different sizes and in different parts of Europe (Fig. 2).

The potential of green–blue urban areas for cooling cities is generally well established, and has been studied using direct observations^15,16^, remote sensing^17^ or modelling based approaches^18,19^. The regulation of local urban air temperatures by such areas can increase thermal comfort and decrease health risks related to urban heat island (UHI) effects^20,21^ for urban populations. The UHI effects relate to often-observed higher ambient air temperatures in urban environments compared to their close surroundings^20,21^. The spatial extents of cities in this study are then considered according to their respective administrative unit definitions.

The investigation focuses on urban realization of this ecosystem service because the proportion of the global human population living in urban areas is steadily rising^22^, and cities are critical for both climate change mitigation and societal adaptation to warming^23,24^. For adaptation, cities need to handle exacerbated urban warming by UHI effects and provide livable environments for their residents while avoiding detrimental consequences from competing development interests^25,26^. The UHI effects emphasize the importance of local climate regulation as an essential urban ecosystem service, the actual realization of which depends on city function and form, with the latter including the spatial distribution of green–blue urban areas, as well as temporal changes in this by growing urbanization. The degree to which such growth leads to replacement of moist soils and vegetative cover with paved and impervious surfaces also affects urban surface energy and radiation balances^27^, and associated land surface temperatures at local human scale, although the relationship with air temperature is complex^27^. For example the proportion of vegetation in a particular area will regulate the resulting ratio of sensitive to latent heat flux (known as Bowen ratio), which will in turn affect properties of the urban climate^27^.

In reality, a city’s climate consists of a variety of smaller-scale microclimates, which can be modified and leveraged through deliberate design^20^. This emphasizes the importance of good city planning^28^, including for conservation, restoration, and construction of new urban green–blue areas^29,30^. Such areas can provide various services to urban populations, e.g., urban flood mitigation^12^ and more general health^31^ and well-being^32^ benefits, including cooling required to mitigate UHI effects. The latter can be achieved, e.g., by enhanced latent heat flux associated with higher evapotranspiration from green areas and evaporation from blue areas. Through the flow of air and its lateral heat advection, green–blue urban areas can also cool surrounding built parts of the city that would commonly have a demand for such ecosystem service of local climate regulation^2^. How to measure and predictively quantify the zones of influence of such air cooling by green–blue areas is still a challenging research question, but such zones are reported to be in the range of several hundred meters^29,33,34^.

The aim of the indicators developed and used in this study is to quantify actual realized urban ecosystem service supply in terms of its fulfillment of some actual demand for that ecosystem service of the urban human population. Over each city, such realization and associated indicator values depend both on local conditions (such as natural land-cover areas that can supply the considered ecosystem service) and overall urban form and spatial configuration of the natural and built areas in the urban landscape. At larger scales spanned by multiple cities (such as those over Europe studied in this paper, Fig. 2), the quantitative indicators can be used to detect main ecosystem service realization patterns, similarities and differences among cities. This is done by quantifying indicator statistics across the cities, and assessing ecosystem service realization patterns in terms of how these statistics depend on city characteristics, or associated country or sub-region characteristics, such as population density or socio-economic measures like Human Development Index (HDI) and GDP per capita.

A few studies have evaluated spatial dependencies of ecosystem services^35,36^ and mostly focused on multiple services in a specific study area. Our comparative multi-city study aims instead at revealing possible overarching statistical patterns of the spatially dependent ecosystem service of local climate regulation, and its realization in and across European urban systems. While this urban ecosystem service is important per se, the dependence of its realization on spatial proximity to green–blue areas may also provide useful guidance for further study of other urban ecosystem services that depend on the spatial distribution of green–blue areas and their proximity to human needs within cities^2,12,32^.

Previous multi-city explorations of urban socio-economic growth and human-made infrastructure have revealed and quantified various statistical cross-city patterns^37–39^. Our study hypothesizes that such patterns may also emerge in the cross-city statistics of ecosystem service realization indicators related to green–blue city areas and their provision to urban populations. Identification of such quantitative ecosystem service indicator patterns can increase fundamental understanding of urban ecosystem service conditions, as well as projection capabilities for changes in these conditions under city growth, e.g., in terms of population density, HDI, and GDP per capita.

To explore and test the main study hypothesis, we compile and synthesize for all 660 European cities (Fig. 2) high-resolution datasets for city morphology (e.g., land cover) and bio-physical characteristics (e.g. degree of imperviousness, vegetation type and vegetation density), based on previous study reports of the relevance of these parameters for the ecosystem service of local climate regulation^2,12^, along with city-scale measures of human population, city area, and resulting population density ratio (Supplementary Table 1). Using these data, we evaluate and map total potential ecosystem service supply and demand in each city (Figs. 1, 2, Supplementary Figures 1–3, Methods), and further apply a model of radially decaying ecosystem service supply and demand realization at 20 m resolution (Supplementary Figure 2–3, Methods) to also account for the spatial influence reach of local climate regulation from each location in the city. Furthermore, for comparative multi-city analysis, we quantify a set of directly comparable ecosystem service realization indicators for each city (explained further below) and their resulting statistics across all 660 cities over Europe, and comparatively for cities in different European countries and sub-regions.

### Indicator definitions and calculations

For each of the 660 cities, we consider and calculate two basic metrics of urban ecosystem service realization: the ratio of realized to potential ecosystem service supply (Rs/Ps), and the ratio of realized to potential ecosystem service demand (Rd/Pd). For each discretized city pixel within a city, we first calculate its local net potential ecosystem service supply (Ps) or demand (Pd) directly from the urban morphology and bio-physical data (Supplementary Figure 1). For each net supply pixel, we further calculate (as illustrated bottom right in Supplementary Figure 2) that pixel’s ecosystem service realized supply contributions to the surrounding net demand pixels within its spatial influence radius (top, Supplementary Figure 2). Analogously, for each net demand pixel, we calculate the contributions to fulfilling (realizing) its ecosystem service demand from the surrounding net supply pixels that have that net demand pixel within their spatial influence radius. For each pixel of any type, we thus calculate its realized ecosystem service supply Rs or demand Rd in relation to its potential net local supply Ps or demand Pd, respectively (Supplementary Figure 2; see also Supplementary Figure 3 and Supplementary Information for further calculation and mapping details). We further calculate comparative indicators of city-average relative realized ecosystem service supply and demand, Rs/Ps and Rd/Pd, respectively, from the sums of local Rs, Rd, Ps and Pd over all pixels in the city. The city-average supply indicator Rs/Ps thus quantifies the average degree of realized (actually used) ecosystem service supply from all green–blue areas over the whole city (left in Fig. 1). Analogously, the city-average demand indicator Rd/Pd quantifies the average degree of realized (actually fulfilled) ecosystem service demand over each city (right in Fig. 1). For further cross-city comparison, we also calculate indicators for how large area fraction of total city area has a relatively high degree of ecosystem service supply and demand realization, respectively. Local Rs/Ps ≥ 0.5 and Rd/Pd ≥ 0.5 are then selected as illustrative thresholds for such relatively high degree of ecosystem service supply and demand realization, respectively, with the area fractions calculated from the number of pixels with Rs/Ps ≥ 0.5 or Rd/Pd ≥ 0.5 relative to the total number of pixels in each city.

Based on the power-law relationships with population density results found for both previous city-average and city-fraction indicators of ecosystem service realization, we also have an opportunity to project indicator values for future scenarios of changed population density, as

1 $$r_{i} = \frac{Ri}{{Pi}} = Ai \cdot \left( {PD} \right)^{\beta i} \le 1$$

where index i = d represents demand and i = s supply. Furthermore, for city-average indicators, Ri and Pi represent realized and potential ecosystem service, respectively, while for area-fraction indicators, they represent city area with high degree of ecosystem service realization (≥ 0.5) and total city area, respectively. The constraint of $r_{i} \le 1$ is due to the upper limit of Ri ≤ Pi for both indicator types, with Ai the scale factor and βi the exponent of a power law relationship r_i_ with population density (denoted PD). Based on Eq. (1), a relative measure of ecosystem service realization effectiveness can be estimated from the demand fulfillment ($r_{d}$) relative to the supply use ($r_{s}$), as:

2a $$Effectiveness = \frac{{r_{d} }}{{r_{s} }} = \frac{{Ad \cdot \left( {PD} \right)^{\beta d} }}{{As \cdot \left( {PD} \right)^{\beta s} }} = \frac{Ad}{{As}}PD^{{\left( {\beta d - \beta s} \right)}}$$

with

2b $$r_{d} = Ad \cdot \left( {PD} \right)^{\beta d} \quad if\quad r_{d} \le 1,\,\,\,\,r_{d} = 1\quad otherwise$$

2c $$r_{s} = As \cdot \left( {PD} \right)^{\beta s} \quad if\,r_{s} \le 1,\,\,\,r_{s} = 1\quad otherwise.$$

## Results

### Comparative ecosystem service realization indicators and their cross-city statistics over Europe

**Figure 3:**
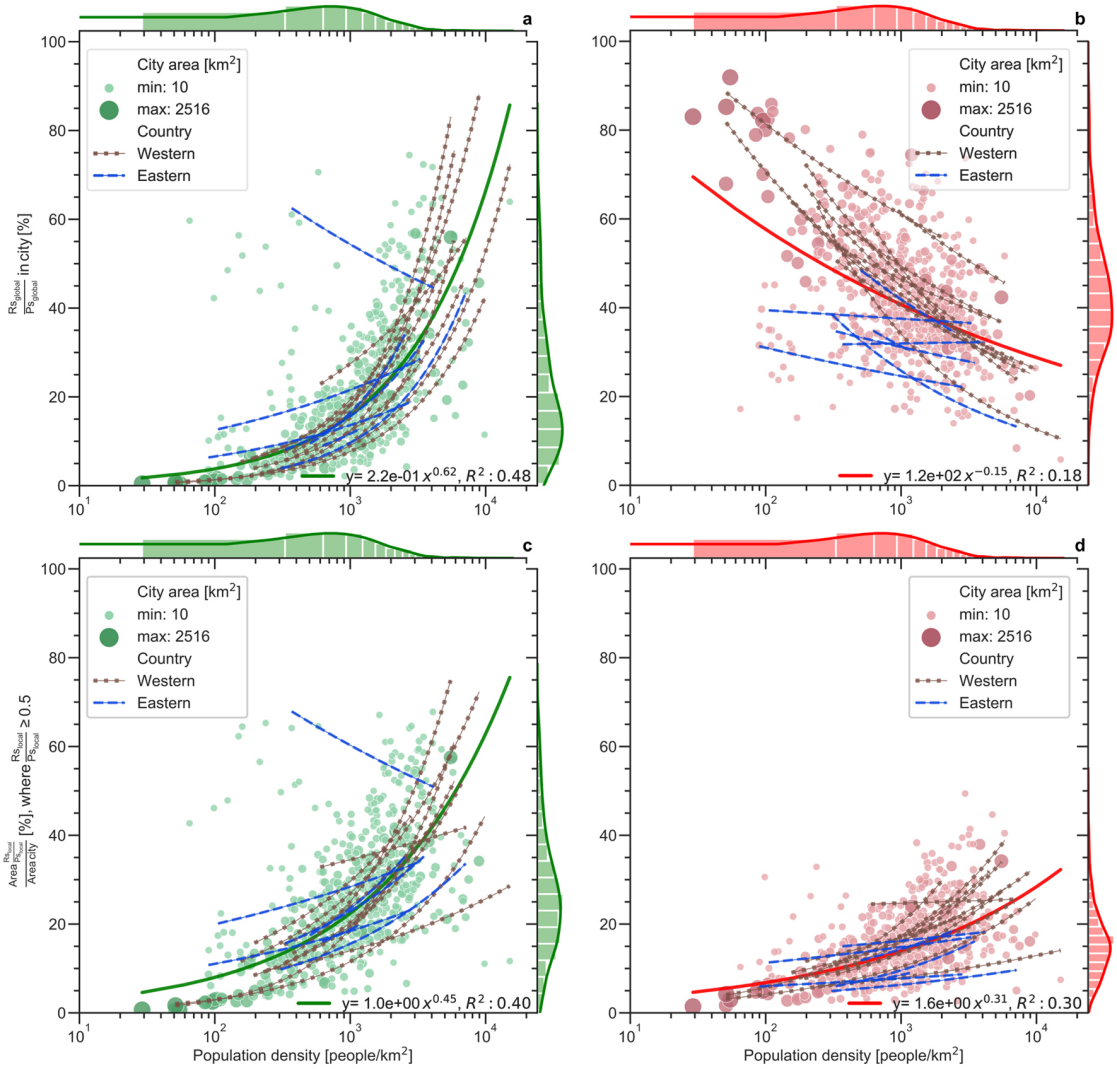
Comparative quantification of ecosystem service realization in cities across Europe. For all 660 European cities studied (green and red symbols and associated solid lines for supply and demand, respectively), and cities within separate countries (blue dashed and brown filled-symbol lines for eastern and western European countries, respectively), indicator results are shown for: city-average realized to potential ecosystem service (**a**) supply (Rs/Ps) and (**b**) demand (Rd/Pd); and city-area fraction with high degree (≥ 0.5) of local ecosystem service (**c**) supply and (**d**) demand realization. Solid lines show best power-law fit for all cities (whole-Europe results), with associated equation y and coefficient of determination R^2^ values also given in each panel, along with histograms of the total number of observations in various population density and fraction intervals. Furthermore, the blue dashed and brown filled-symbol lines in each panel show the best power-law fit for cities within each eastern and western European country, respectively; associated scaling factor A, power-law exponents β, and coefficients of determination R^2^ for each country and western and eastern European sub-region are listed in Supplementary Table 2, while full city-average and area-fraction results for each country are illustrated in Supplementary Figures 6 and 7, respectively.

Resulting indicator values for city-average realized supply Rs/Ps exhibit sub-linear power-law increase with increasing city population density population density across all studied European cities (solid green line, with power-law exponent β = 0.62, Fig. 3a). Corresponding indicator values for city-average realized demand Rd/Pd further exhibit sub-linear decline with increasing population density (solid red line, β = − 0.15, Fig. 3b). In combination, these indicator results imply that European cities with higher population density commonly use an increasingly larger part of their total potential ecosystem service supply to fulfill a decreasing part of their total ecosystem service demand.

In accordance with city-average supply Rs/Ps (Fig. 3a), city area fraction with relatively high ecosystem service supply realization (local Rs/Ps ≥ 0.5) also increases with increasing population density (Fig. 3c). That is, cities with larger (smaller) city-average Rs/Ps also tend to have larger (smaller) area fraction with high (≥ 0.5) local ecosystem service supply realization. For ecosystem service demand fulfillment, however, the area-fraction behavior (Fig. 3d) differs from that for city-average Rd/Pd (Fig. 3b). While city-average demand fulfillment decreases, the city area fraction with high demand fulfillment (Rd/Pd ≥ 0.5) increases with increasing population density. More specifically, this city area fraction is overall small (< 20%) for small-medium population density (up to 10^3^ per km^2^) and increases to around 30–40% as population density approaches the largest city values of the order of 10^4^ per km^2^. This indicator result implies that European cities with higher population density tend to make better use of their local green–blue areas for fulfilling ecosystem service demand in just some parts of the city while they have less ecosystem service demand fulfillment on average over the whole city.

The decrease in city-average ecosystem service demand fulfillment (Rd/Pd) with increasing population density (Fig. 3b) reflects a combination of increasing population pressure (increasing potential demand, Pd) on the available ecosystem service supply resource (the potential supply, Ps), and progressive decrease of this resource (Ps) by replacement of green–blue areas with other urban land uses under city growth. Natural infrastructures provide greater ecosystem services when located closer to many beneficiaries, however land also becomes more expensive with increasing population pressure. Conserving or restoring these ecosystem services in these conditions is thus costly, and in direct competition with other land uses^40^. The potential ecosystem service supply of a city can thus reach the full capacity limit of Rs = Ps (i.e., the total ecosystem service potential supply) with no additional Ps left to meet the growing demand (Pd) of a higher population density, even without any decrease in the ecosystem service supply (Ps). Additionally, if the urbanization process for accommodating a higher population density decimates the green–blue areas of a city, the supply (Ps) will also decrease, so that the upper capacity limit Rs = Ps becomes smaller and even less sufficient for meeting the growing ecosystem service demand (Pd).

In the process of population density increase, previous low-density areas (e.g., characterized by ≤ 30% soil sealing) with high ecosystem service demand fulfillment (high local Rd/Pd,  Fig. 3d) may shift into high-density areas that use (i.e., realize) most or all of their (likely decreasing) potential ecosystem service supply. Alternatively, low-density urban areas may mainly expand into surrounding green–blue areas (forest, open fields, wetland areas after drainage) while still maintaining their low-density characteristics. This latter type of expansion, which includes relatively affluent low-density residential areas, but potentially also light industries or institutions for example, may explain the increase seen in area fraction with high ecosystem service demand fulfillment for higher population density (Fig. 3d). In contrast, common densification leads to overall increase in city fraction with high ecosystem service supply use (Fig. 3c) and thereby to decrease in city-average ecosystem service demand fulfillment (Fig. 3b).

In general, the indicator values for both city-average ecosystem service realization and city area fraction with high ecosystem service realization correlate better with population density than either total population size (Supplementary Figure 4) or city area (Supplementary Figure 5). Between the latter two, indicator values exhibit better power-law fit with city area than with total population size.

### Country-wise cross-city comparison

Differences in ecosystem service realization for cities with similar population density may depend on variations, e.g., in city history, planning practices, and socio-economics. Such factors may be more similar for cities within a country than between countries, which is why we further also quantify and compare country-wise indicator patterns that may reveal such influences (blue dashed and brown filled-symbol lines in Fig. 3).

Power-law fits of indicator values with population density are better (have higher coefficient of determination, R^2^) for the country-wise results than for all cities over Europe (see Supplementary Table 2, Supplementary Figures 6–7). For city-average indicators, resulting mean R^2^ is 0.64 for supply (Rs/Ps) and 0.39 for demand (Rd/Pd) in country-wise fitting, while R^2^ is 0.56 and 0.20 for fitting to all cities (591) in the individually represented countries (with ≥ 8 cities each), and 0.48 and 0.18 for fitting to all cities (660) , respectively. For area-fraction indicators, mean R^2^ in country-wise fitting is on average 0.52 for supply and 0.38 for demand, while R^2^ is 0.45 and 0.32 for fitting to all cities in individually represented countries, and 0.4 and 0.3 for fitting to all cities, respectively.

On average, the country-wise fitted power-law exponents (β) are largely consistent with those fitted to all cities over Europe. However, distinct parameter differences emerge in β values for cities of western and those of eastern European countries (Fig. 3, Supplementary Table 2). For city-average ecosystem service realization, country-wise fitting yields average β for western cities of 0.76 for supply (Rs/Ps) and − 0.23 for demand (Rd/Pd) (with average R^2^ of 0.79 and 0.52, respectively), while average β for eastern cities is 0.45 for Rs/Ps and − 0.13 for Rd/Pd (with average R^2^ of 0.45 and 0.19, respectively). For area fractions with high ecosystem service realization (≥ 0.5), western cities have average β of 0.49 for supply and 0.34 for demand (with average R^2^ of 0.63 and 0.52, respectively), while average β for eastern cities is 0.25 for supply and 0.22 for demand (with average R^2^ of 0.38 and 0.19, respectively).

Overall, western cities (brown filled-symbol lines in Fig. 3b) exhibit higher levels of city-average ecosystem service demand fulfillment (Rd/Pd) than eastern cities (blue dashed lines). However, the results are more mixed and more dependent on population density for city-average ecosystem service supply realization (Rs/Ps, Fig. 3a), and for area fractions with high ecosystem service realized supply (local Rs/Ps ≥ 0.5;  Fig. 3c) or demand (local Rd/Pd ≥ 0.5; Fig. 3d). Furthermore, rather than just looking at cities as they are now, a key question explored further in the following is what the statistical patterns of the ecosystem service realization indicators imply for evolving cities and their ecosystem service realization effectiveness under population density growth.

### Population density based projection of ecosystem service realization and its effectiveness

**Figure 4:**
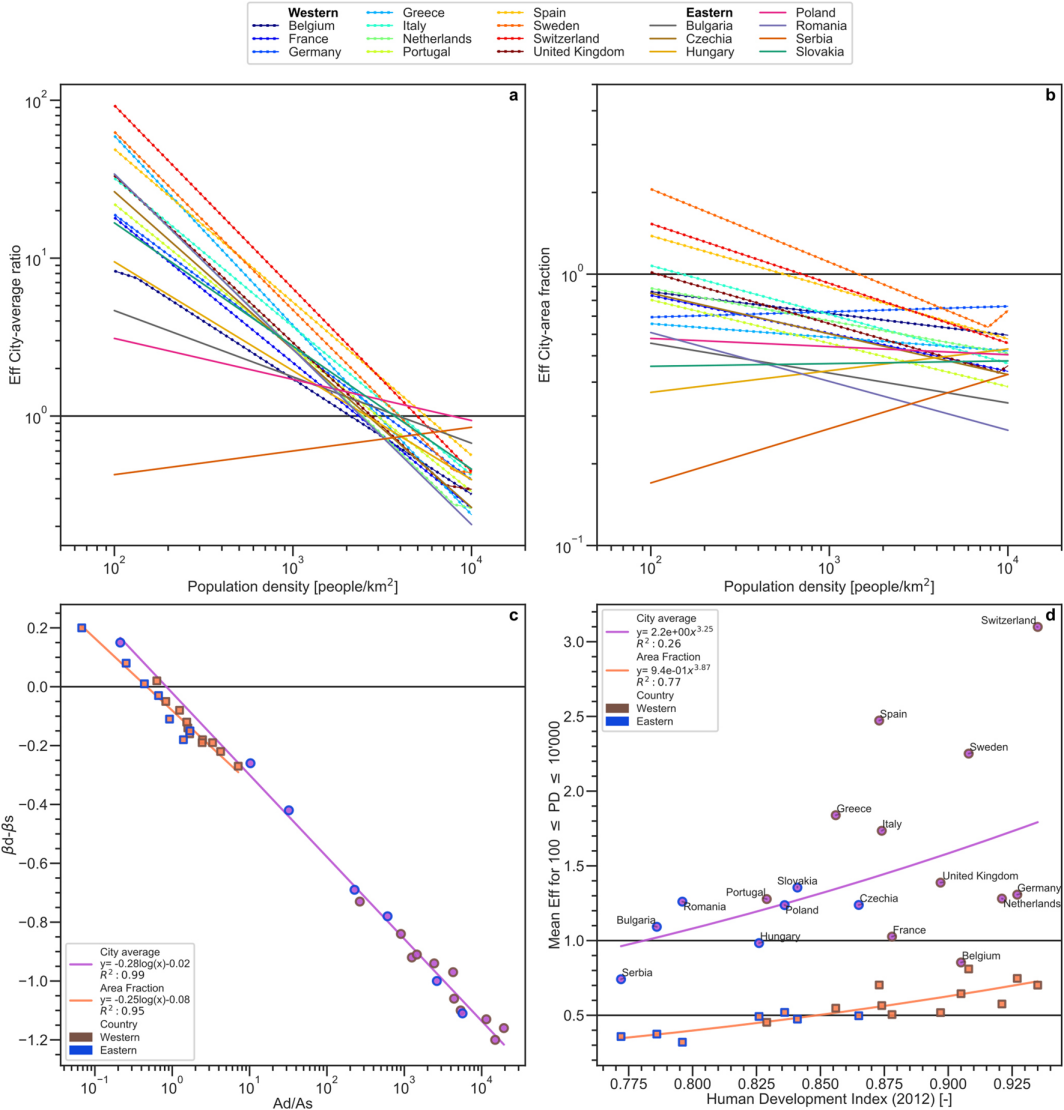
Relationships of effectiveness measure for individual eastern and western European countries. For each individual country (listed in Supplementary Table 2), effectiveness results are shown for the: (**a**) city-average and (**b**) area-fraction indicators of ecosystem service realization; (**c**) co-variation of effectiveness exponent (β_d_ − β _s_) and scale factor (Ad/As); and (**d**) co-variation of average effectiveness (for 100 ≤ population density ≤ 10,000) and Human Development Index (HDI) for each country. Solid and dashed lines in panels (**a**, **b**) show effectiveness values for eastern and western countries, respectively. Purple and orange lines/symbols in panels (**c**, **d**) distinguish city-average ratio of realized to potential ecosystem service and city-area fraction with high degree (≥ 0.5) of local ecosystem service metrics, respectively, with blue and brown symbol outlines showing eastern and western countries respectively, and solid lines showing best log/power law fits for all countries (associated regression equation y and coefficient of determination R^2^ values are also given in each panel). Supplementary Figure 8 further illustrates results corresponding to those in panel d, but for GDP per capita instead of HDI, and Supplementary Figures 9–10 illustrate the co-variation with HDI and with GDP per capita, respectively, of the various exponents β and scale factors A included in the effectiveness expression.

The resulting effectiveness Eq. (2), a measure of demand fulfillment relative to supply use, also shows a power-law relationship with population density (Fig. 4a,b), with exponent (β_d_ − β_s_) and scale factor Ad/As (the index d representing demand, and the index s supply respectively). Exponents and scale factors further emerge as mutually correlated based on the available multi-city data (Fig. 4c).

The country-wise quantification of effectiveness for city-average ecosystem service realization over a relevant range of 100 ≤ population density ≤ 10,000 per km^2^ (Fig. 4a) shows mostly an effectiveness > 1 for cities with relatively low population density up to around 2000–4000 per km^2^. That is, the relative city-average demand fulfillment is in these cases is mostly greater than the relative city-average supply use; Serbia is an exception in exhibiting an effectiveness < 1 for this range of relatively low population density. Overall, higher effectiveness levels emerge for western than eastern cities. However, results mix and converge to generally low effectiveness ≈ < 1 as population density approaches and exceeds 5′000 per km^2^. At the high population density limit, the three countries with highest effectiveness are eastern, including Serbia, Poland and Bulgaria, while the country with the lowest effectiveness is also eastern, Romania. Serbia is also an exception in exhibiting increasing city-average effectiveness for increasing population density; this is further also the only country with realized supply Rs/Ps that decreases for increasing population density (Supplementary Figure 6), due to small green–blue area extent within or near the local administrative boundaries of the Serbian low-density cities (leading to low Ps value and high Rs/Ps).

The effectiveness pattern for area fraction with high local demand fulfillment relative to the area fraction with high local supply realization shows mostly low values of effectiveness ≈ < 1 across the whole range of 100 ≤ population density ≤ 10,000 per km^2^ (Fig. 4b). These low effectiveness values imply some inefficiency in that the area fraction with high demand fulfillment is smaller and increases less with population density than the area fraction with high supply realization. For population density≈ < 1000 per km^2^, three western countries (Sweden, Switzerland, Spain) are exceptions in having an effectiveness ≈1–2, i.e., an area fraction with high demand fulfillment that is about as large or (particularly for Sweden) even up to a factor 2 larger than that with high supply realization. Furthermore, across the whole population density range, three eastern countries (Serbia, Hungary, Slovakia) and one western country (Germany, which also includes former eastern cities) are exceptions in that their average effectiveness increases for higher population density (i.e., their area fraction with high demand fulfillment increases more steeply than that with high supply realization as population density increases); also for these countries, however, effectiveness remains overall low, < 1, over the population density range.

In general, the power-law exponent (β_d_ − β_s_) for relative effectiveness effectiveness declines (logarithmically) with increasing scale constant Ad/As (Fig. 4c). That is, the mainly western countries with the highest effectiveness levels for low population density also have the steepest decreases in effectiveness for increasing population density. Even with these steeper decreases, however, mean effectiveness levels are on average (over the whole urban population density range) greater for western than eastern countries. Noting these and other main result differences between cities in western and eastern European countries, and knowing that average socio-economic and other development conditions also differ between these European sub-regions, we explore in the following if and how some measures of these conditions, like HDI^41^ and/or GDP per capita^42^ (Supplementary Figure 8), correlate with the effectiveness levels.

Results show effectiveness correlation with both HDI (Fig. 4d) and GDP per capita (Supplementary Figure 8), with the fitted β and A power-law parameters differing but the explanatory power (R^2^ values) being similar for both of these socio-economic measures. Supplementary Figures 9–10 further show more or less similarly logarithmic and power-law regressions between the variations of the β and A parameters in effectiveness, respectively, and those of HDI (Supplementary Figure 9) and GDP per capita (Supplementary Figure 10). Overall, these results indicate that socio-economic variations reflected similarly in HDI and GDP per capita may underlie some main parts of the variations seen in ecosystem service realization effectiveness.

In particular, the data-given goodness of fit (R^2^) of the effectiveness power-law relationship with HDI (and GDP per capita) is considerably higher for area-fraction (orange in Fig. 4d) than city-average conditions (purple in Fig. 4d). The difference indicates that scenarios of growth in HDI (and GDP per capita) may be particularly useful for projection of how the overall effectiveness level of ecosystem service realization [based on Eq. (2)] and its quantification for area fraction with high ecosystem service realization, applicable to relatively low-density city parts, may develop under such growth. For city-average ecosystem service realization, scenarios of population density growth should be more useful for projection of its possible development, based on country-wise power-law relationships as expressed in Eq. (1), and in particular for cities in western European countries where the explanatory power of these relationships is especially high (Supplementary Table 2). These statistical relationships do not conclusively determine causal mechanisms, but the data-given quantitative results are still relevant, useful, and worthy of further research to resolve underlying causality.

## Concluding discussion

Across many hundreds of European cities with widely different population density population density (as well as total population and area sizes), the results of this study show some robust power-law relationships of relative ecosystem service realization and its effectiveness (fulfilled demand per realized supply). This applies in particular to country-wise fitted relationships of city-average ecosystem service realization with population density for western European cities, and to the relationship of overall effectiveness level with HDI (and GDP per capita) for low-density urban areas with high ecosystem service realization.

The comparative quantitative indicator results developed and used in this study reveal these key distinctions between various ecosystem service realization indicators and their manifestations in western and eastern European cities. The latter distinctions may reflect ecosystem service realization impacts of different histories, socio-economic evolutions, and urban planning traditions and developments in the western and eastern parts of Europe. Distinguishing and understanding the many factors leading to current ecosystem service form and functioning of these cities is a highly complex endeavor. Additional work and comparisons with spatial properties of urban systems, such as the degree of sprawling and compactness, would be valuable in further work. Nevertheless, the western-eastern divergence revealed in this study implies mostly higher city-average ecosystem service demand fulfillment in western than eastern European cities. However, this fulfillment and its effectiveness per realized ecosystem service supply decreases more with increasing population density in the western than the eastern cities. For more densely populated European cities, demand fulfillment and its effectiveness converge and mix across countries. This indicates that planning and building strategies and practices in densely populated European cities are more similar and play more important roles for ecosystem service realization and effectiveness than country-related differences.

City-scale results depend on city boundary definition, and no clear consensus exists on how to define a city^39^. As an example of possible definition shifts and their result implications consider, e.g., extension of a city’s boundaries to also include large sparsely populated commuting areas. This would increase city area, insignificantly increase total population, and decrease average population density. City-average realization of ecosystem service supply Rs/Ps would then decrease with the decreasing population density by such addition of large area with few people leading to greater increase in potential than realized supply. Furthermore, city-average realization of ecosystem service demand Rd/Pd would increase with such population density decrease due to greater increase in realized than in potential demand by adding new area with few people. In both cases, the shift directions in ecosystem service realization would still remain consistent with the data-given relationships of city-average ecosystem service realization with population density, which would thus be relatively robust to such city boundary changes.

Nevertheless, the discussed boundary and causality questions are important and remain open, calling for further research for their resolution, with particular focus on cities with high population density and their more mixed results among cities in different countries. The relationships identified and quantified here, and their translation into predictive Eqs. (1), (2) can still be useful in indicating possible evolution trajectories for urban ecosystem service realization in European cities under different growth scenarios. Moreover, the approach developed for this quantification can be relevant and useful also for other urban ecosystem services^12,31,32^ that depend on green–blue urban areas and spatial distances from them for realization of their benefits. More generally, rapid developments in big data availability and analysis methods provide new opportunities for further advancements in combined city and ecosystem service realization science that can reveal predictive quantitative relationships, and best urban planning strategies and practices for various urban socio-economic, climate, and environmental conditions and their changes.

## Methods

### Data preparation and interpretation

To assess the extent of potential ecosystem service supply and demand and their realization, we considered 660 cities in the European Union, West Balkans, and EFTA (European Free Trade Association). These represent a variety of urban forms, population counts, topography, climate, and vegetation characteristics. They were used here for potential ecosystem service supply and demand quantification, as outlined and explained further in Supplementary Figure 1 and following scoring methods developed and applied previously^2,12^. We used the highest resolution spatial products available, from standardized and open sources to ensure reproducibility, and with coverage across our set of cities. We used the Urban Atlas 2012 product^43^ to determine the baseline land cover class of functional urban area (FUA) at high resolution (20 m). The original Urban Atlas contains 24 unique land cover classes (17 for artificial surfaces). However, four categories (‘Green urban areas’, ‘Industrial, commercial, public, military and private units’, ‘Airports’, and ‘Sports and leisure facilities’) are too broad to represent accurately the physical cover in urban areas. We further used the dominant leaf type (DLT) product^43^ to separate forested areas from the ‘Green urban areas’ class. The latter class thus only refers to managed urban grass and bush vegetation types. For the three remaining categories, we extracted pixels with imperviousness > 20% using the imperviousness degree (IMD) product^43^. This threshold was used to distinguish constructed areas, which retained their original classification (meaning that ‘Industrial, commercial […]’, ‘Airports’ and ‘Sports and leisure facilities’ classes referred only to the artificial parts of such categories). Remaining unclassified vegetated areas were then further separated, using the DLT product, into ‘forests’ or ‘green urban areas’ land cover classes.

When land cover information was not available around city boundaries, we complemented the data with the CORINE 2012 land cover data^43^ to calculate ecosystem service realization near these boundaries (original resolution of 100 m, downscaled to 20 m for the analysis), including also sea and ocean areas potentially omitted in Urban Atlas 2012. The city boundaries from the Urban Atlas 2012 product were then used as final city delineations for calculating individual city statistics. These boundaries represent a local administrative unit where at least 50% of the population lives in one or more urban centers, with the latter identified as groups of grid cells with population density of at least 1500 inhabitants/km^2^ and collectively a population of at least 50,000 inhabitants. It does not include the less densely populated city commuting zone, in other words the surrounding travel-to-work areas of a city (which if included forms a functional urban area, for larger cities)^44^. This allows us to focus on cities’ capacity to provide the ecosystem service of interest based on a common typology (urban areas), rather than also include commuting zones which may include suburbs, towns or rural areas, depending on the city considered. Furthermore, we used the 2015 population density dataset from the Socioeconomic Data and Application Center (SEDAC)^45^ to calculate the average population density in each city, as well as the average population density of relevant urban fabric land cover classes. The total population in city delineations was approximately 178 millions, compared to 339 millions in functional urban areas (which includes cities). Additional datasets used in the study were the tree cover density (TCD) product^43^, and normalized difference vegetation index (NDVI)^46,47^. The final land cover resolution is 20 m, while the NDVI and population density datasets have resolution of 250 m and 30 arc-seconds (roughly 1 km resolution at the equator) respectively. NDVI statistics based on land cover resolution were produced for the assessment matrix (Supplementary Figure 1). Although we are confident in the average per land cover class statistics on NDVI, it is important to note that these measured values for small features and/or small areas of land cover types can be affected by the surrounding environment (similarly for population density). NDVI measures are also dependent on the inherent temporality of the physical cover (in particular for agricultural land cover classes). Comparison with other indicators (such as degree of imperviousness) thus helps the interpretation. Population count and population density values were averaged over the larger Urban Atlas 2012 city boundary. See Supplementary Table 1 for further details on the datasets and their uses.

### The ecosystem service of local climate regulation

Energy from solar radiation and anthropogenic activities is absorbed by the urban system, and is ultimately partitioned between local air warming (by convection and radiation), moisture evaporation, and heat storage in surface materials and near the surface belowground^27^. Vegetated (green) and water-covered (blue) spaces provide varying ecosystem services. The ecosystem service of interest in this study was their potential to mitigate the UHI effect and dampen increasing temperatures and extreme events from future climate change. One of the most important features of the cooling effect of blue/green areas is the evaporation and transpiration process^27,29^, whereby energy is absorbed and released as latent heat rather than sensible heat^27^. We used a supply/demand framework^12,13^ to quantify the potential of natural areas to contribute to local air temperature reduction, and the subsequent potential need for this service by the urban population (Supplementary Figure 1). Potential supply (Ps) was defined here as ‘the hypothetical maximum capacity for service provision’ of a particular ecosystem to human well-being, regardless of its actual consumption. Similarly, potential demand (Pd) was defined here as ‘the hypothetical maximum service need’ of humans in a particular area, regardless of its fulfillment. These definitions are a result of our spatially explicit approach, which differentiate potential and actual services based on spatial connections, conceptualized based on earlier studies^8,12,48^. We used a look-up matrix approach linking land cover classes with a relative 0–5 scale scoring system^12^, as a starting point to quantify both Ps and Pd. This evaluation provided a simple way to obtain comparable rankings and ranges for both the natural and human side of the ecosystem service equilibrium.

We scored each land cover class potential supply based on average vegetation density, amount of artificial surfaces, vegetation types, tree density, and presence of water. A denser and/or larger vegetation amount (which also depends on vegetation types and structure) and increased water area will generally provide larger cooling benefits. We scored potential demand based on average land cover class imperviousness and population density (see Supplementary Table 1 and Fig. 1 for details on the datasets and scores). The higher the imperviousness density, the higher the amount of energy stored in artificial surfaces. Moreover, demand exists only if human beneficiaries exist (in varying urban environments), with thus a higher demand for outdoor heat comfort in locations with higher population concentrations. These score values represented our estimates, based on satellite product indicators and our hypothesis, on the average (Europe-wide) capacity of a land cover category to provide or consume the service studied. As such, we do not model or measure air temperature directly, but rather the assumed influence of natural areas on air temperature and its need by urban population to achieve a better thermal comfort. The scoring system employed was relatively simple, and was applied to each of the 660 cities. The objective was to compare city performance based on the assumption of a similar level of capacity to provide services and/or to fulfill human needs based on land cover typologies, rather than to disentangle regional or local differences in capacities between similar land cover categories. For example, water availability is dependent on larger-scale background climate and precipitation and influences vegetation health and evapotranspiration rates^49^. Landscape irrigation is theoretically possible at the city scale for water-limited environments (to thus reach similar Ps capacity), although the cost would vary between cities. Similarly, average demand in a city is dependent on imperviousness and population concentration, but also on larger-scale current and future climate circumstances^50^. The population adapts to the local climate, with evidence of varying heat tolerance with latitude, but it is uncertain whether it can adapt in time to rapid changes^51^. Demand would thus be more related to temporal shifts in regional temperature than to absolute temperature (which increases along a north–south latitudinal gradient in Europe).

### Ecosystem service supply–demand realization

Ecosystem service provision is spatiotemporally dependent on carrier flows, e.g., on air and water movements (natural carriers) or vehicles (human-made carriers), between areas of service supply and service demand. In particular, local climate at the urban scale is a unique combination of radiative, aerodynamic, thermal, and moisture properties of surrounding constituent surfaces. It is spatially highly variable, so the location in relation to surrounding urban (artificial and natural) elements is important^27^. The term ‘ecosystem service flow’ refers here to the spatial transfer path between supply and demand areas, with realization of the service of local climate regulation thus depending on this local proximal air flow. We defined realized supply (Rs) as ‘the part of the supply actually used’ by service-consuming human beneficiaries in a flow’s area range. Similarly, we defined realized demand (Rd) as ‘the part of the demand actually met’ by service-providing natural areas in a flow’s proximal range. Hence the following relationships exist:

$$Potential \: supply\left( {Ps} \right) = Realized \: supply\left( {Rs} \right) + Remaining \: supply$$

$$Potential \: demand\left( {Pd} \right) = Realized \: demand\left( {Rd} \right) + Remaining \: demand$$

where the Remaining supply (demand) is ‘the part of the supply (demand) not used (met)’. From this simple relationship, it is important to note that Rs ≤ Ps and Rd ≤ Pd, meaning that realization of a service supply and/or demand never exceeds its potential.

Our distance-weighting function was based on an area-weighted average of five declining surface linear functions (100–500 m; see Supplementary Figure 2 for a full graphical explanation). Realization of the service was calculated as:

$$Rs_{p} = \mathop \sum \limits_{i = 1}^{n} Weight_{i} \times Pd_{i}$$

$$Rd_{p} = \mathop \sum \limits_{i = 1}^{n} Weight_{i} \times Ps_{i}$$

where Rs_p_ and Rd_p_ is realized supply and demand, respectively, in pixel *p*, Weight_i_ is the weight in a surrounding pixel *i*, and Ps_i_ and Pd_i_ is potential supply and demand, respectively, in a surrounding pixel *i*. The spatial weight surface function is normalized, with $\sum weights = 1$ in the 500 m radius.

The basic assumption in this approach is that proximity matters, and that the closer supply and demand are connected, the more they will influence each other’s realization. For example, parks or forest systems in this approach (and with the relatively fine pixel resolution used in our present application of it) are not a priori delineated by some idealised distinct border line. They emerge as areas with some relatively high but still varying degree of vegetation (and associated cooling supply) from the actual fine-resolved data. Nonetheless, a size effect does emerge, with larger effective cooling ranges for larger park/forest systems that both fulfil more demand and reach demand in and around their wider boundary area zones than those of smaller park/forest areas. This applies up to the limit of 500 m range considered in this study from any outermost pixel in the relatively wide boundary area zones of large park/forest systems. In further research, this approach could be extended to explore various effects, such as the size-range effect, e.g., by exploring different and variable pixel-influence ranges, but such further exploration is outside the scope of the present multi-city comparative study.

Our focus with this methodology is to expand on spatial supply–demand realization frameworks with a relatively simple calculation approach that can readily be used for comparative multi-city analysis at the still necessary high spatial resolution for studying each urban system. Use of more advanced and detailed microclimatological modeling applied to each city would not have been feasible for the large number of cities included in this study. Moreover, such detailed modeling would also require calibration to local variables (such as local air temperatures), the possible non-uniqueness (equifinality) of which would introduce additional uncertainties in need of quantification and further implication assessment for each and comparatively across the numerous studied cities. Each modelling approach comes with its own limitations, and we prioritized in this study to use the relatively simple approach allowing us to readily obtain comparable results for and across these numerous cities. By this approach, all calculations and analyses could be performed at a baseline pixel resolution of 20 m. All data manipulation, calculations, and analyses were performed using ArcGIS 10.5^52^ and the Python programming language^53^.

## Supplementary Information

Supplementary Information.

## Data Availability

The authors declare that all data supporting the findings of this study are available within the paper and its supplementary information files.

## Code availability

The code that underlies and supports the findings of this study is available from the corresponding author upon reasonable request.
