# Supplementary material for: Comparative quantification of local climate regulation by green and blue urban areas in cities across Europe
Source: Sci Rep. 2021 Dec 13;11:23872. doi: 10.1038/s41598-021-03140-y (PMC8669022; doi:10.1038/s41598-021-03140-y)
Supplement: Supplementary file 1 — Supplementary Information. [file 41598_2021_3140_MOESM1_ESM.docx]

**Supplementary Information**

**Comparative quantification of local climate regulation by green-blue urban areas in cities across Europe**

Romain Goldenberg, Zahra Kalantari, Georgia Destouni

**Supplementary material: list of material provided**

**Supplementary Table 1 |** Spatial datasets used in the study

**Supplementary Figure 1 |** Assessment matrix for potential ecosystem service (ES) supply and demand.

**Supplementary Figure 2 |** Schematic illustration of the approach to quantifying the realization of local climate regulation

**Supplementary Figure 3 |** Detailed exemplification of mapping and quantification of potential and realized supply/demand for local climate regulation in the city of Stockholm, Sweden.

**Supplementary Figure 4 |** Comparative quantification of ecosystem service (ES) realization with total population size across Europe.

**Supplementary Figure 5 |** Comparative quantification of ecosystem service (ES) realization with city area across Europe.

**Supplementary Table 2 |** Statistics of indicator relationship with population density (PD)

**Supplementary Figure 6 |** Individual per-country trends of city-average ecosystem service (ES) realization.

**Supplementary Figure 7 |** Individual per-country trends of city-area fraction ecosystem service (ES) realization.

**Supplementary Figure 8 |** Effectiveness measure (Eff) co-variation (for 100 ≤ population density ≤ 10‘000) with GDP per capita for each country

**Supplementary Figure 9 |** Effectiveness measure (Eff) components trends with Human Development Index (HDI)

**Supplementary Figure 10 |** Effectiveness measure (Eff) components trends with GDP per capita.

**Supplementary Note 1 |** Quantification example for Stockholm (Supplementary Fig. 3), in the city boundary.

**Supplementary Table 1 | Spatial datasets used in the study.**

| **Name**  **[*original name*]** | **Resolution / Accuracy** | **Temporal reference** | **Institute** | **Used for** |
| --- | --- | --- | --- | --- |
| **Urban Atlas LCLU 2012 CityBoundary ^43^** | Geometric resolution: 0.25 ha,  Positional accuracy: 5 m | 2012 | CLMS (Copernicus Land Monitoring Service) | City delineation |
| **Urban Atlas LCLU 2012 ^43^** | Geometric resolution: 0.25 ha,  Positional accuracy: 5 m | 2012 | CLMS | Physical cover creation |
| **Corine Land Cover**  [*CLC2012*] **^43^** | Min. mapping unit / width: 25 ha / 100m,  Geometric accuracy: better than 100 m | 2012 | CLMS |  |
| **Forests Dominant Leaf Type**  [*DLT-2012-20m-Full*] **^43^** | 20 m | 2012 | CLMS |  |
| **Tree Cover Density**  [*TCD-2012-20m-Full*] **^43^** | 20 m | 2012 | CLMS |  |
| **Water & Wetness**  [*WAW-2015-20m-Full*] **^43^** | 20 m | 2015 | CLMS |  |
| **Imperviousness Density**  [IMD-2012-20m-Full] **^43^** | 20 m | 2012 | CLMS | Physical cover creation and assessment matrix statistics |
| **Population Density** [*Gridded Population of the World, Version 4 (GPWv4): Population Density Adjusted to Match 2015 Revision UN WPP Country Totals, Revision 11*] **^45^** | 30 arc-second  (~1 km at the equator) | 2015 | NASA Socioeconomic Data and Applications Center (SEDAC) | Population statistics and assessment matrix statistics |
| **NDVI**  [*MOD13Q1 MODIS/Terra Vegetation Indices 16-Day L3 Global 250m SIN Grid V006*] **^46^** | 250 m | 1^st^ June to 1^st^ September, 2011-2013  (7 × 16days × 3years) | LPDAAC | NDVI statistics for background information (main Fig.1) and assessment matrix statistics |
| **NDVI**  [*MYD13Q1 MODIS/Aqua Vegetation Indices 16-Day L3 Global 250m SIN Grid V006*] **^47^** | 250 m | 1^st^ June to 1^st^ September, 2011-2013  (6 × 16days × 3years) | LPDAAC |  |

**Note.** DAAC: Distributed Active Archive Center; MODIS: Moderate Resolution Imaging Spectroradiometer; NASA EOSDIS: National Aeronautics and Space Administration Earth Observing System Data and Information System; NDVI: Normalized Difference Vegetation Index. Only “good data” NDVI pixel retrievals are used, identified from the corresponding MOD13Q1/MYD13Q1 pixel reliability layers. Based on this selection, we produce a mean pixel value dataset over the whole time period considered for NDVI (combining Aqua and Terra datasets), which is used in the present study.


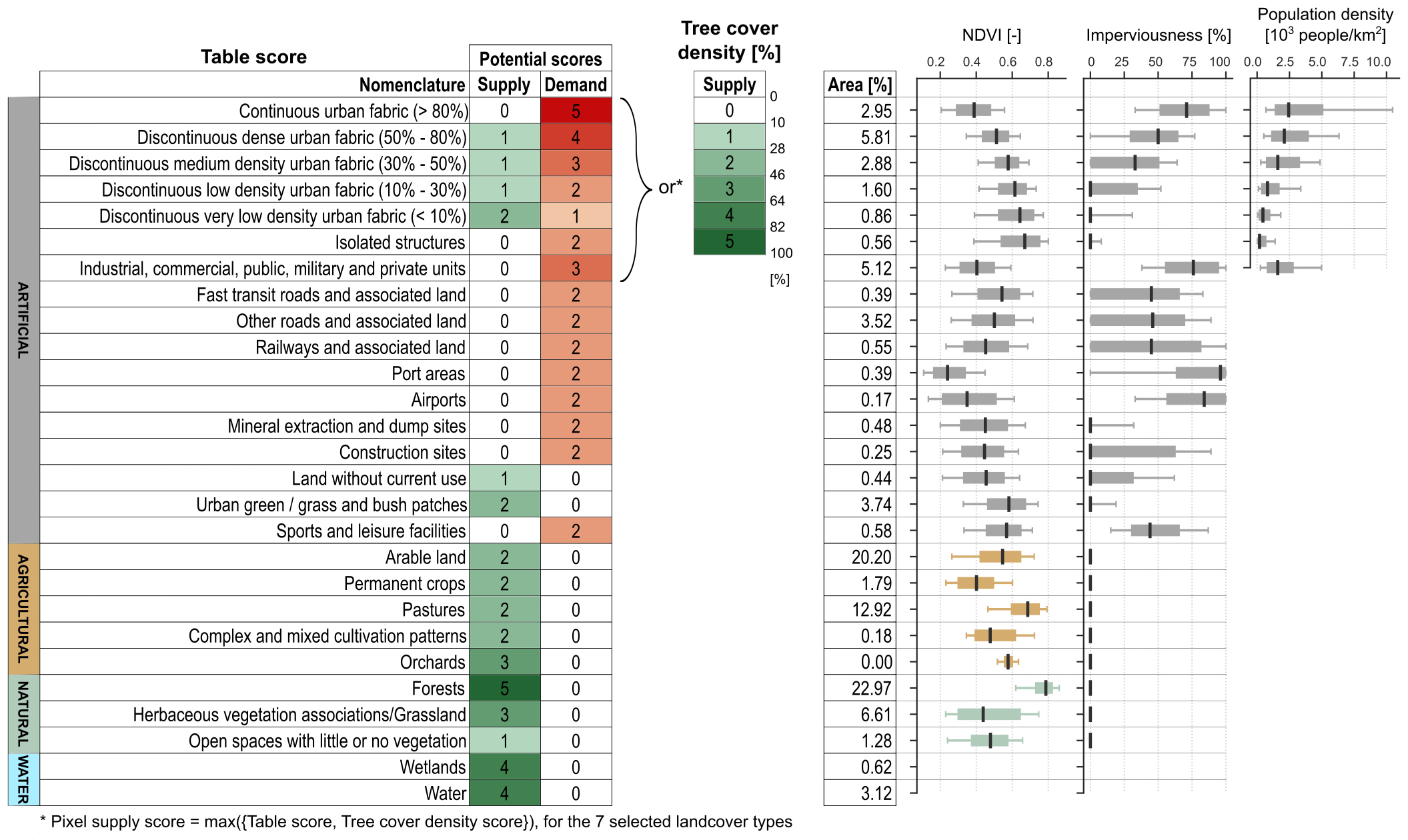


**Supplementary Figure 1 | Assessment matrix for potential ecosystem service (ES) supply and demand.** Estimated potential supply (Ps) and potential demand (Pd) of the ES of local climate regulation is displayed for different land covers within a city. A score of 0 signifies no relevant potential ES supply capacity (Ps) or demand (Pd), while a score of 5 signifies the highest potential supply capacity/demand level. For urban fabric land cover types, the Ps baseline is determined from the relative ratio of vegetation/imperviousness. Since these categories include various vegetation types for different countries, we supplemented the urban fabric scores with tree-cover density scores for supply (with ultimate Ps then being the maximum of the two scores). Area proportions are calculated for each land cover type across the 660 cities studied. The boxplots of normalized difference vegetation index (NDVI), imperviousness degree, and population density are also calculated for each land cover class over the whole dataset (boxes indicate 25%, median, and 75% percentile, whiskers indicate 10% and 90% percentiles). Note that satellite products have varying resolution so, in particular for NDVI (250 m), measured values for small features and/or small areas of land cover types can be affected by the surrounding environment. See Supplementary Table 1 for further dataset details, and Supplementary Fig. 2 for more methods details.


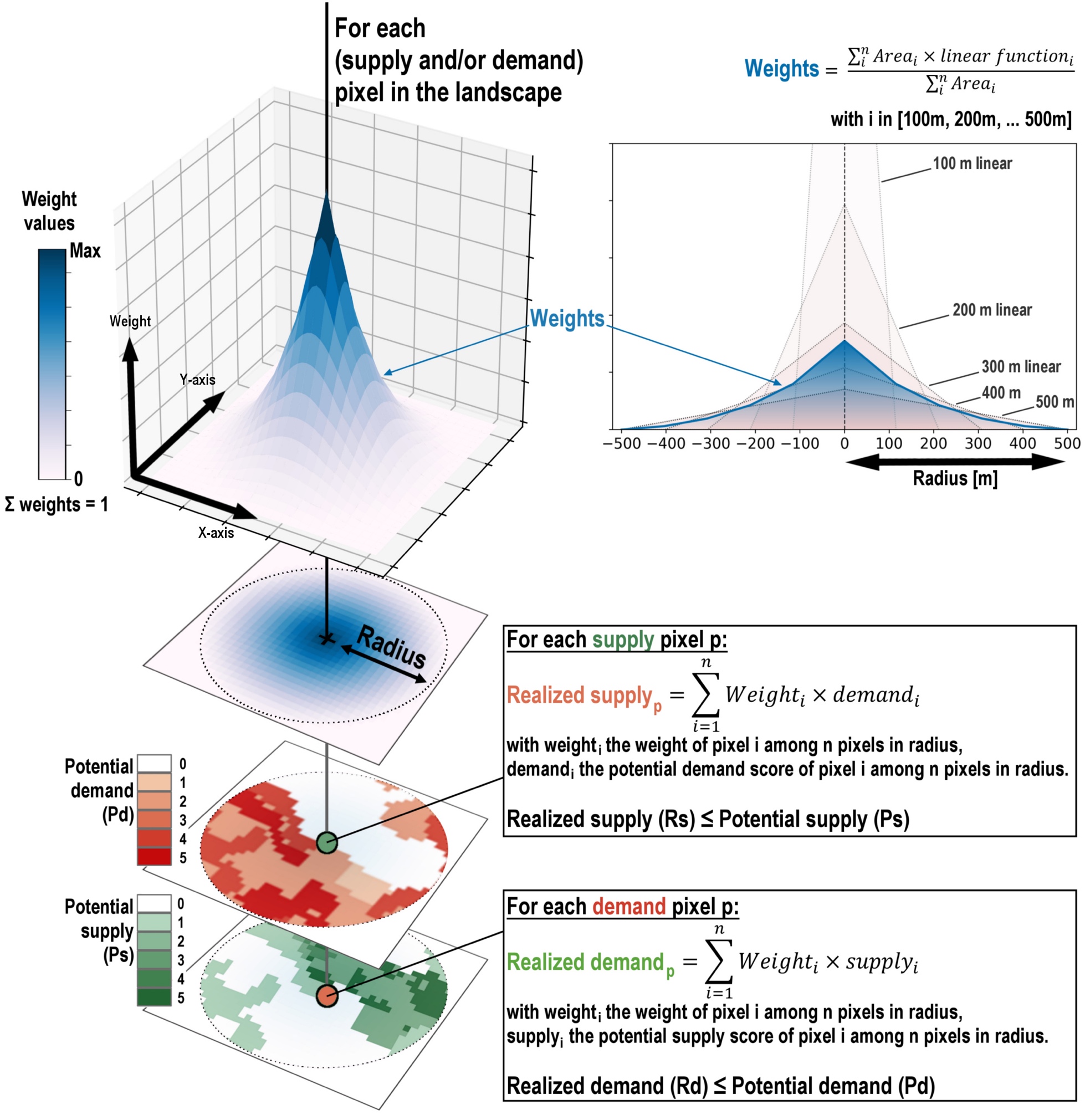


**Supplementary Figure 2 | Schematic illustration of the approach to quantifying the realization of local climate regulation.** Spatial influence zones of such regulation (air cooling) by green-blue urban areas are reported to be in the range of several hundred meters^21,25,26^. In this quantification of urban realization characteristics of the ecosystem service (ES) of local climate regulation, we consider and account for radial ES decay over such distances (top right panel). See further Methods for more detailed approach explanations, and Supplementary Fig. 3 and Supplementary Note 1 for more detailed description and exemplification of city-wise quantification and mapping of different metrics for the city of Stockholm (Sweden).


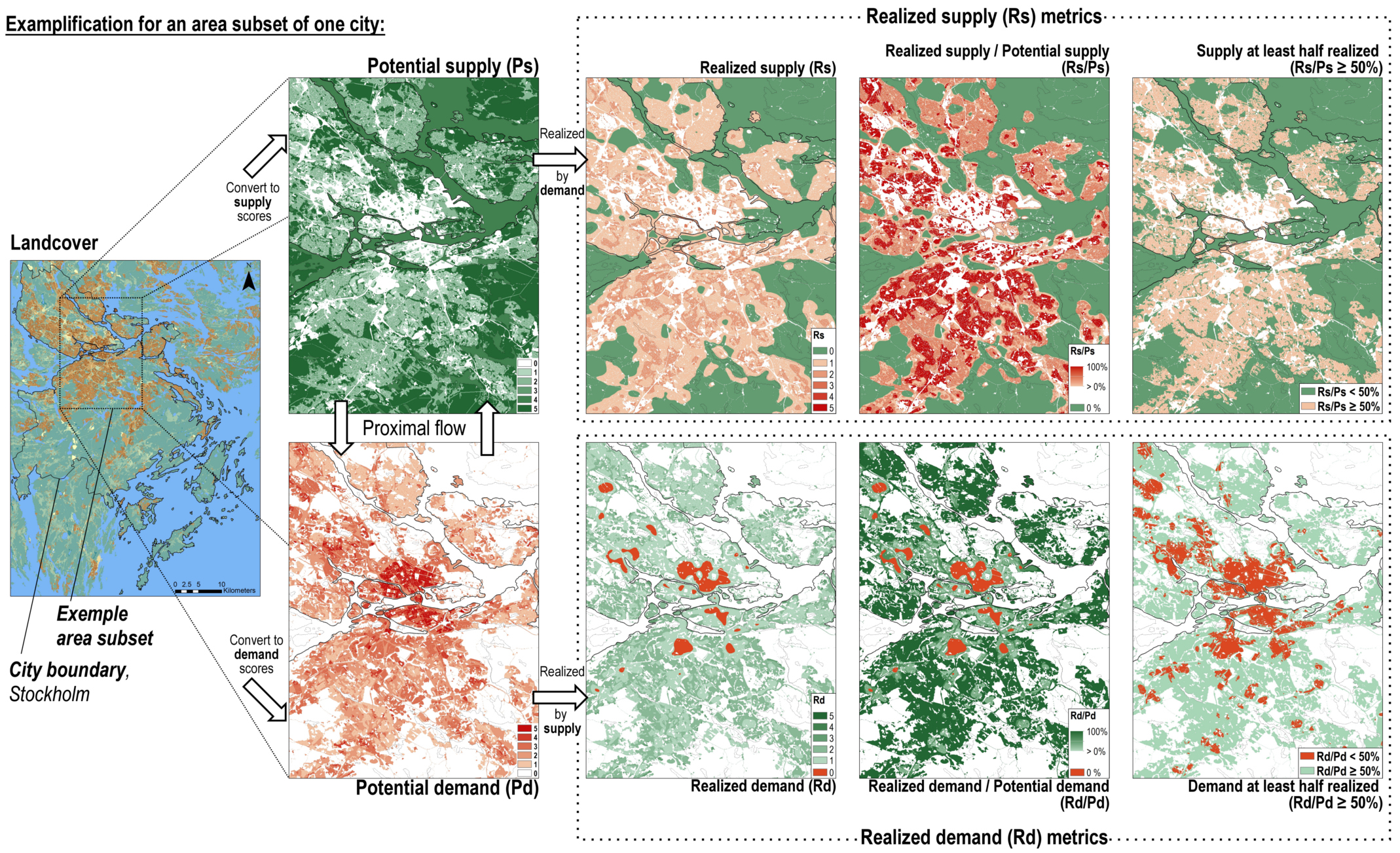
 **Supplementary Figure 3 | Detailed exemplification of mapping and quantification of potential and realized supply/demand for local climate regulation in the city of Stockholm, Sweden.** Landcover at 20 meters resolution is created based on the material in Supplementary Table 1, with subsequent conversion to Ps and Pd maps according to the classification set in Supplementary Fig. 1. We then calculate and map Rs and Rd based on the flow dependence model described in Supplementary Fig. 2. Rs (Rd respectively) shows the absolute value of realized supply (demand respectively) per pixel. Rs/Ps (Rd/Pd respectively) shows the degree of realization to initial potential supply (demand respectively) per pixel. Rs/Ps ≥ 50% (Rd/Pd ≥ 50% respectively) shows the area where pixels have their supply (demand respectively) at least half realized. A quantification example of the reported metrics for Stockholm is provided in Supplementary Note 1.


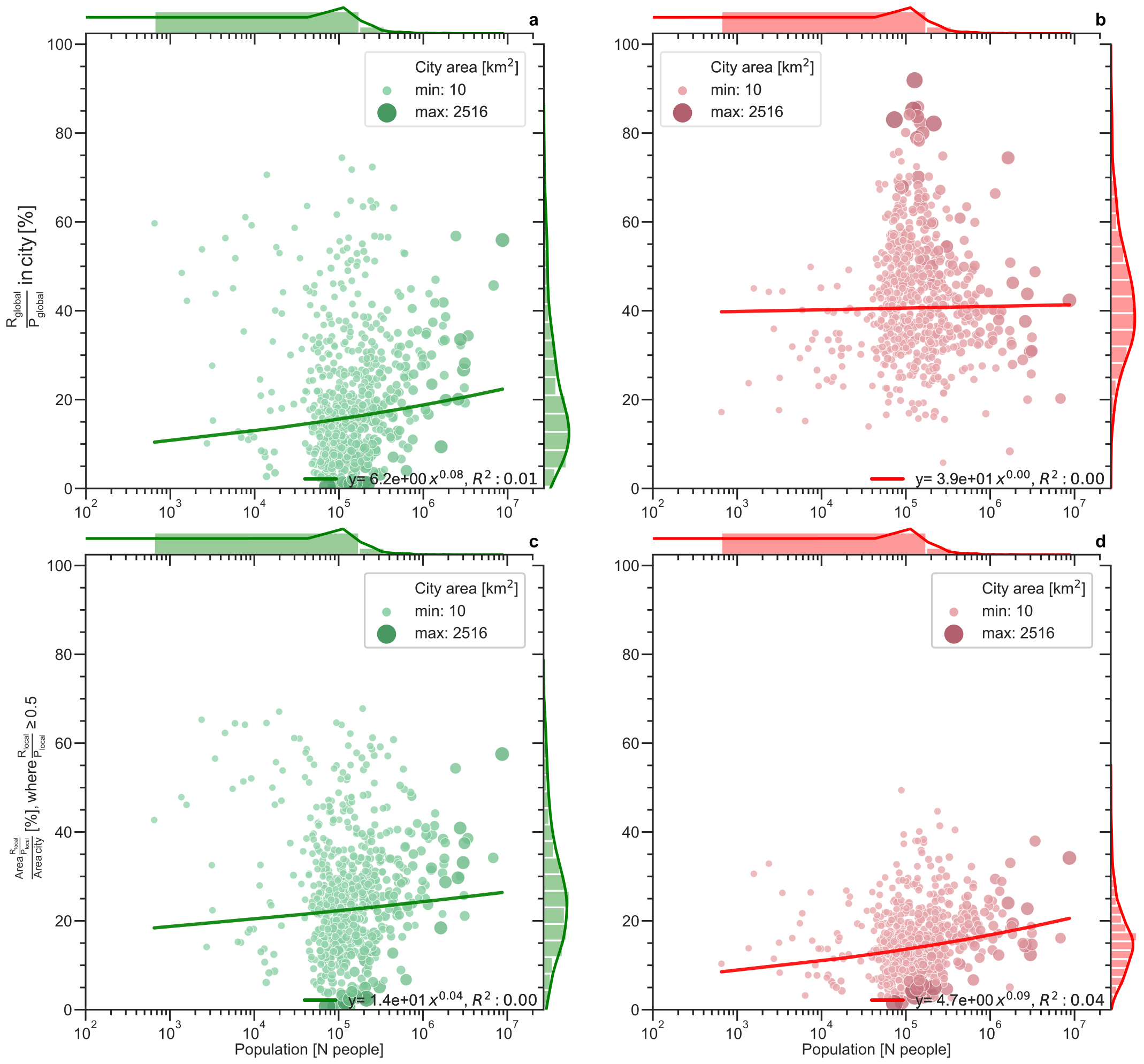


**Supplementary Figure 4 | Comparative quantification of ecosystem service (ES) realization with total population size across Europe**. For all 660 European cities studied (green and red symbols and associated solid lines for supply and demand, respectively), results are shown for: city-average ratio of realized to potential ES (A) supply (Rs/Ps) and (B) demand (Rd/Pd); and city-area fraction with high degree (≥0.5) of local ES (C) supply and (D) demand realization. Solid lines show best power-law fit for all cities (whole-Europe results), with associated equation y and coefficient of determination R^2^ values also given in each panel, along with histograms of the total number of observations in various population size and area fraction intervals.


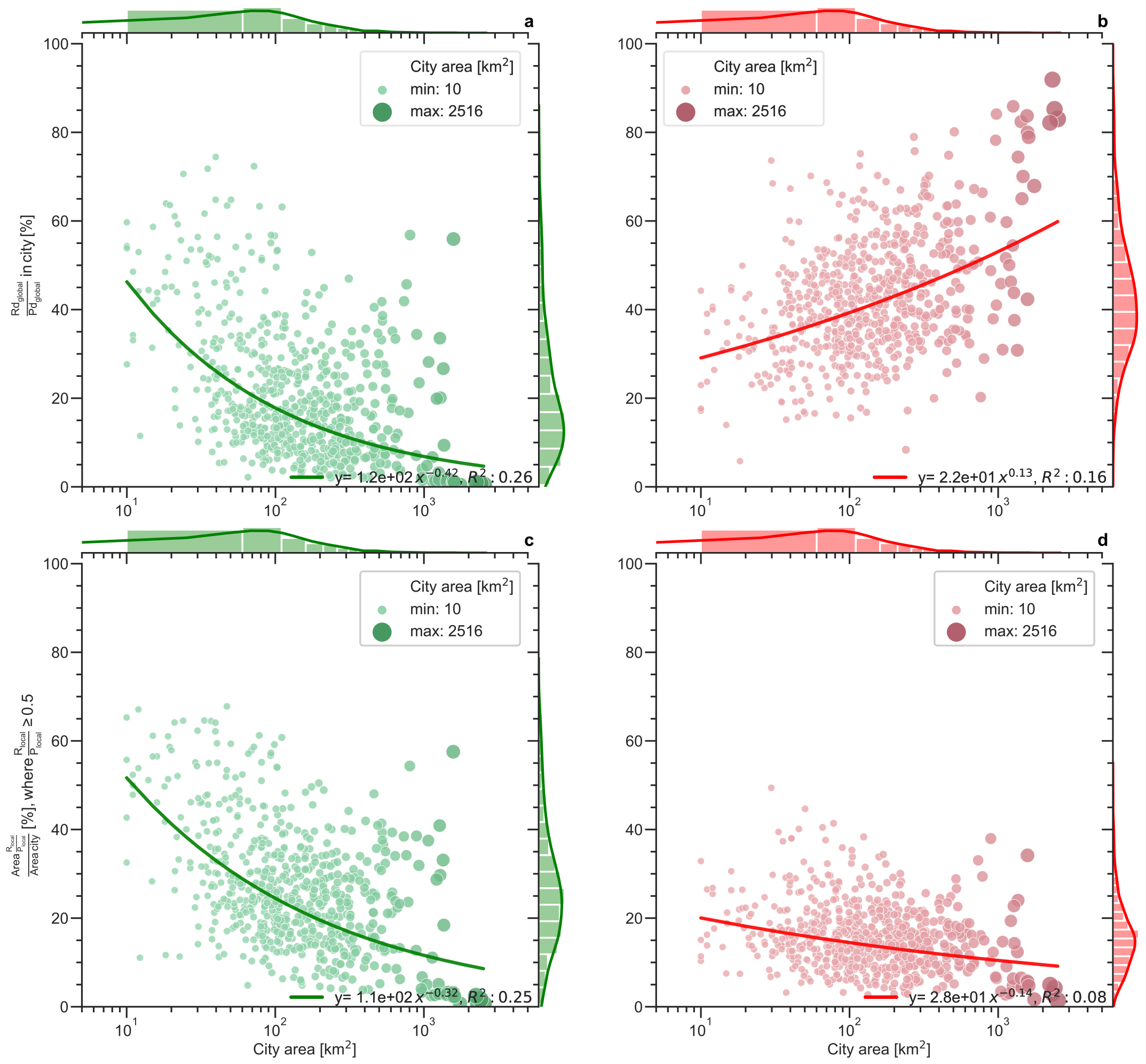


**Supplementary Figure 5 | Comparative quantification of ecosystem service (ES) realization with city area across Europe**. For all 660 European cities studied (green and red symbols and associated solid lines for supply and demand, respectively), results are shown for: city-average ratio of realized to potential ES (A) supply (Rs/Ps) and (B) demand (Rd/Pd); and city-area fraction with high degree (≥0.5) of local ES (C) supply and (D) demand realization. Solid lines show best power-law fit for all cities (whole-Europe results), with associated equation y and coefficient of determination R^2^ values also given in each panel, along with histograms of the total number of observations in various city area and area fraction intervals.

**Supplementary Table 2 | Statistics of indicator relationship with population density (PD).** Parameters of best power-law fit: scale factor A, exponent β and coefficient of determination R^2^. N is number of cities in each country, and only countries with N≥8 are represented individually in the table^#^.

|  |  | **City-average ratio** | | | | | | **City-area fraction** | | | | | |
| --- | --- | --- | --- | --- | --- | --- | --- | --- | --- | --- | --- | --- | --- |
|  | **Cities**  **count** | **Supply** | | | **Demand** | | | **Supply** | | | **Demand** | | |
|  |  | **As** | **βs** | ***R^2^*** | **Ad** | **βd** | ***R^2^*** | **As** | **βs** | ***R^2^*** | **Ad** | **Βd** | ***R^2^*** |
| **All cities (whole Europe)** | 660 | 0.22 | 0.62 | *0.48* | 116 | -0.15 | *0.18* | 1.01 | 0.45 | *0.40* | 1.63 | 0.31 | *0.30* |
| **Cities in countries**  **with N≥8** | 591 | 0.15 | 0.67 | *0.56* | 128 | -0.16 | *0.20* | 0.86 | 0.47 | *0.45* | 1.45 | 0.32 | *0.32* |
| **Western European**  **Countries** |  |  |  |  |  |  |  |  |  |  |  |  |  |
| Belgium | 11 | 2.30 | 0.36 | *0.53 *** | 611 | -0.37 | *0.70 ** | 17.6 | 0.10 | *0.07 **** | 21.9 | 0.02 | *0.00 **** |
| France | 82 | 0.16 | 0.69 | *0.74* | 204 | -0.23 | *0.47* | 1.19 | 0.45 | *0.61* | 1.90 | 0.31 | *0.41* |
| Germany | 91 | 0.13 | 0.70 | *0.61* | 121 | -0.14 | *0.14* | 1.51 | 0.40 | *0.51* | 0.96 | 0.42 | *0.37* |
| Greece | 9 | 0.05 | 0.77 | *0.95* | 681 | -0.43 | *0.76 ** | 2.26 | 0.26 | *0.32 **** | 1.87 | 0.21 | *0.40 **** |
| Italy | 67 | 0.10 | 0.69 | *0.53* | 255 | -0.25 | *0.46* | 0.56 | 0.51 | *0.48* | 1.39 | 0.33 | *0.39* |
| Netherlands | 31 | 0.03 | 0.90 | *0.84* | 166 | -0.20 | *0.67* | 0.52 | 0.53 | *0.74* | 0.80 | 0.41 | *0.79* |
| Portugal | 11 | 0.20 | 0.65 | *0.83* | 288 | -0.26 | *0.77* | 2.02 | 0.39 | *0.63 ** | 3.39 | 0.23 | *0.51 *** |
| Spain | 63 | 0.04 | 0.75 | *0.81* | 191 | -0.22 | *0.57* | 0.20 | 0.59 | *0.69* | 0.66 | 0.40 | *0.57* |
| Sweden | 12 | 0.01 | 1.00 | *0.97* | 145 | -0.13 | *0.71* | 0.06 | 0.82 | *0.99* | 0.46 | 0.55 | *0.99* |
| Switzerland | 10 | 0.01 | 0.99 | *0.96* | 195 | -0.17 | *0.31 **** | 0.18 | 0.67 | *0.96* | 0.76 | 0.45 | *0.80* |
| United Kingdom | 47 | 0.04 | 0.89 | *0.90* | 164 | -0.17 | *0.21 ** | 0.30 | 0.64 | *0.88* | 0.73 | 0.45 | *0.46* |
| Mean | 434 | 0.28 | 0.76 | *0.79* | 275 | -0.23 | *0.52* | 2.40 | 0.49 | *0.63* | 3.16 | 0.34 | *0.52* |
| Standard deviation (SD) |  | 0.67 | 0.18 | *0.17* | 190 | 0.09 | *0.22* | 5.10 | 0.20 | *0.28* | 6.27 | 0.15 | *0.27* |
| Coefficient of variation (CV) |  | 2.40 | 0.24 | *0.21* | 0.69 | -0.40 | *0.43* | 2.12 | 0.41 | *0.44* | 1.98 | 0.43 | *0.52* |
| **Eastern European**  **Countries** |  |  |  |  |  |  |  |  |  |  |  |  |  |
| Bulgaria | 17 | 1.53 | 0.32 | *0.34 *** | 49 | -0.10 | *0.16 **** | 3.96 | 0.22 | *0.33 *** | 3.66 | 0.11 | *0.08 **** |
| Czechia | 15 | 0.07 | 0.79 | *0.77* | 182 | -0.21 | *0.33 *** | 1.19 | 0.43 | *0.56 ** | 2.02 | 0.28 | *0.26 **** |
| Hungary | 10 | 0.27 | 0.59 | *0.59 ** | 61 | -0.10 | *0.13 **** | 2.12 | 0.34 | *0.47 *** | 0.54 | 0.42 | *0.50 *** |
| Poland | 58 | 4.25 | 0.24 | *0.16 ** | 44 | -0.02 | *0.01 **** | 9.85 | 0.15 | *0.12 ** | 6.56 | 0.12 | *0.10 *** |
| Romania | 35 | 0.05 | 0.77 | *0.58* | 271 | -0.34 | *0.53* | 1.03 | 0.39 | *0.35* | 1.44 | 0.21 | *0.14 *** |
| Serbia | 14 | 144 | -0.14 | *0.24 **** | 31 | 0.01 | *0.00 **** | 138 | -0.12 | *0.47 ** | 9.32 | 0.08 | *0.04 *** |
| Slovakia | 8 | 0.24 | 0.56 | *0.22 **** | 147 | -0.22 | *0.07 **** | 1.80 | 0.34 | *0.14 **** | 0.78 | 0.35 | *0.11 **** |
| Mean | 157 | 21.4 | 0.45 | *0.45* | 112 | -0.14 | *0.19* | 22.5 | 0.25 | *0.38* | 3.47 | 0.22 | *0.19* |
| SD |  | 53.8 | 0.33 | *0.24* | 90.6 | 0.12 | *0.20* | 50.9 | 0.19 | *0.15* | 3.31 | 0.13 | *0.17* |
| CV |  | 2.51 | 0.74 | *0.53* | 0.81 | -0.88 | *1.06* | 2.26 | 0.76 | *0.40* | 0.95 | 0.58 | *0.92* |
| **All countries Mean** | 591 | 8.50 | 0.64 | *0.64* | 211 | -0.20 | *0.39* | 10.2 | 0.40 | *0.52* | 3.28 | 0.30 | *0.38* |
| **All countries SD** |  | 33.7 | 0.29 | *0.26* | 175 | 0.11 | *0.27* | 32.1 | 0.22 | *0.27* | 5.20 | 0.15 | *0.28* |
| **All countries CV** |  | 3.97 | 0.45 | *0.41* | 0.83 | -0.57 | *0.70* | 3.14 | 0.57 | *0.53* | 1.58 | 0.50 | *0.74* |

**Note.** *** indicates two-sided p value ≥ 0.05, ** p value ≥ 0.01, * p value ≥ 0.001. ^#^ Countries not presented individually in the table (with city count N < 8) include: Albania (N=3), Austria (N=6), Bosnia and Herzegovina (N=5), Croatia (N=5), Cyprus (N=2), Denmark (N=4), Estonia (N=3), Finland (N=7), Iceland (N=1), Ireland (N=5), Kosovo (N=3), Latvia (N=4), Lithuania (N=6), Luxembourg (N=1), Malta (N=1), Montenegro (N=1), Norway (N=6), Republic of Macedonia (N=4) and Slovenia (N=2).


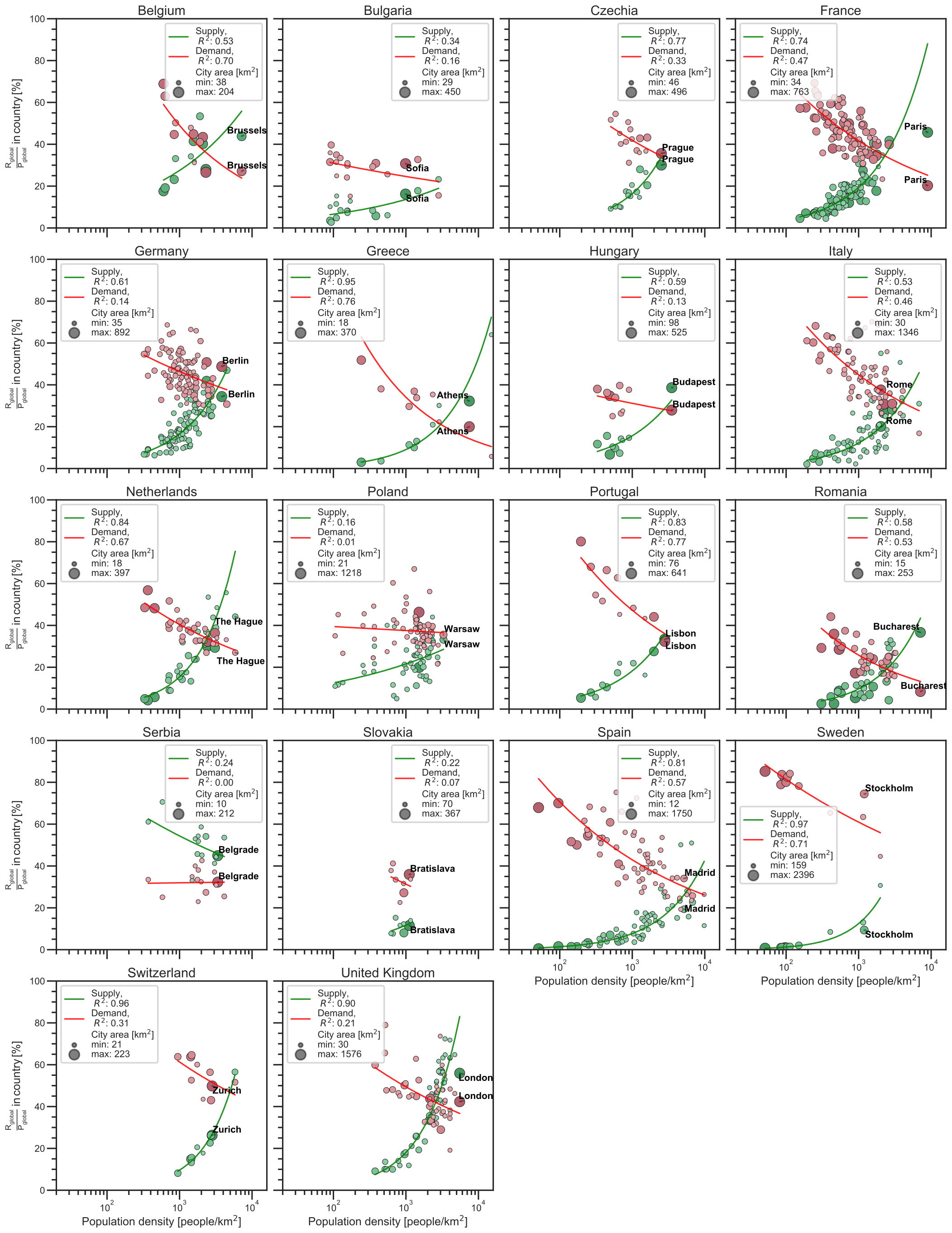


**Supplementary Figure 6 | Individual per-country trends of city-average ecosystem service (ES) realization.** For each country studied (green and red symbols and associated solid lines for supply and demand, respectively), results are shown for city-average ratio of realized to potential ES supply (Rs/Ps) and demand (Rd/Pd). Solid lines show best power-law fit for cities within the country, with associated coefficient of determination R^2^ values also given in each panel (Scale factor A and exponent β are given in Supplementary Table 2). We also highlight here the capital of each individual country.


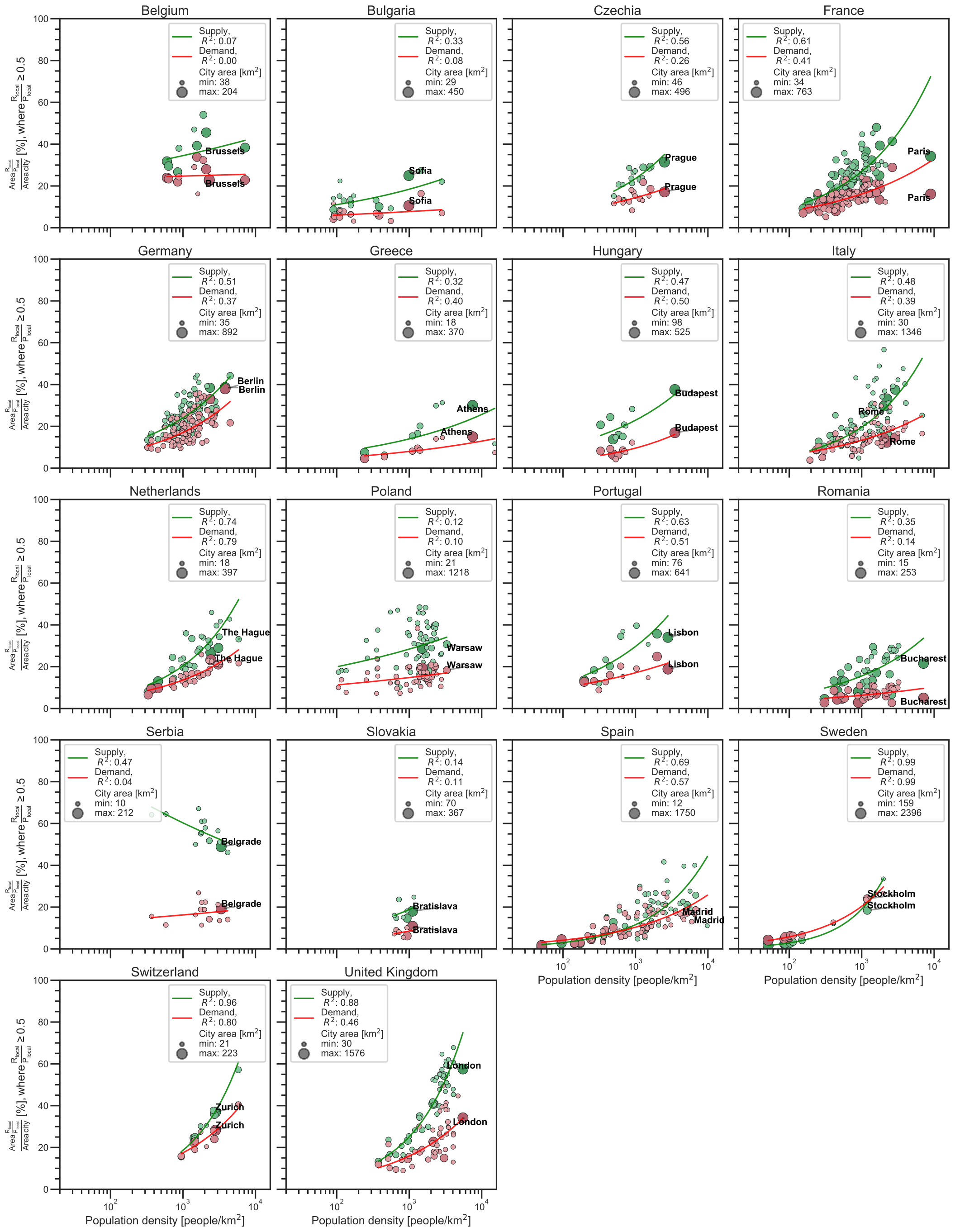


**Supplementary Figure 7 | Individual per-country trends of city-area fraction ecosystem service (ES) realization.** For each country studied (green and red symbols and associated solid lines for supply and demand, respectively), results are shown for city-area fraction with high degree (≥0.5) of local ES supply and demand realization. Solid lines show best power-law fit for cities within the country, with associated coefficient of determination R^2^ values also given in each panel (Scale factor A and exponent β are given in Supplementary Table 2). We also highlight here the capital of each individual country.


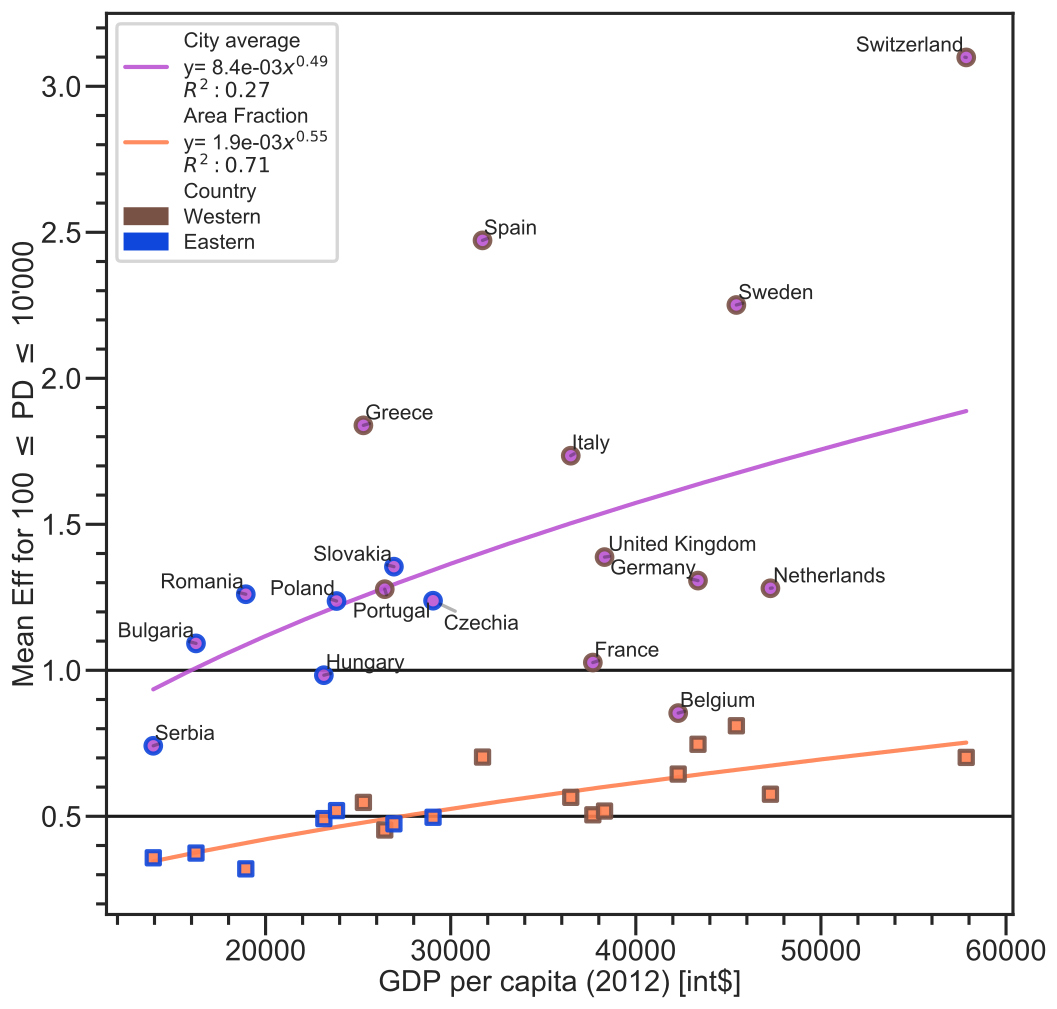


**Supplementary Figure 8 | Effectiveness measure (Eff) co-variation (for 100 ≤ population density ≤ 10‘000) with GDP per capita for each country.** Purple and orange lines/symbols distinguish city-average ratio of realized to potential ES, and city-area fraction with high degree (≥0.5) of local ES metrics respectively, with blue and brown symbol outlines showing eastern and western countries respectively. Solid lines show best power law fits for all countries, with associated equation y and coefficient of determination R^2^ values also given in the panel.


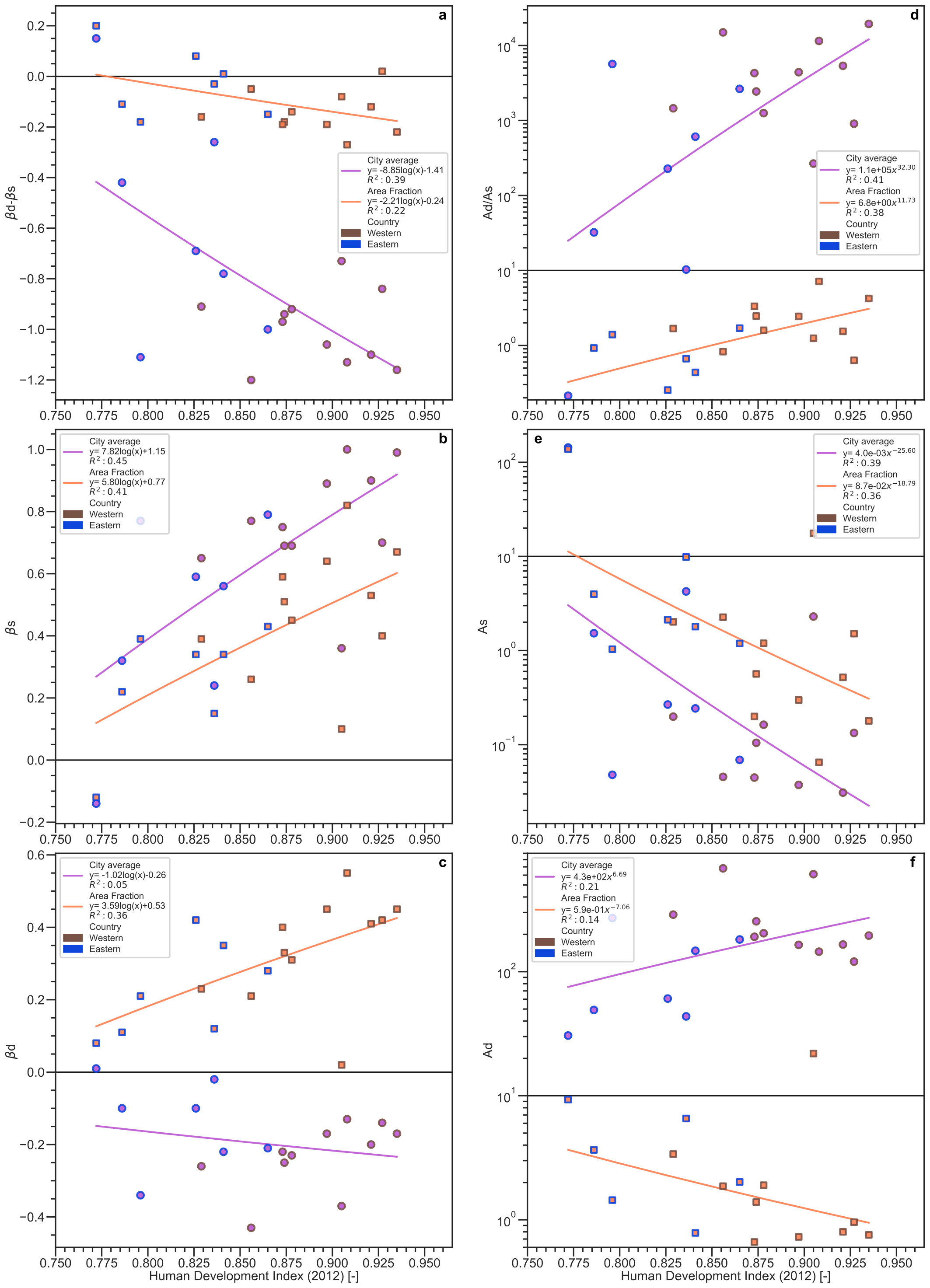


**Supplementary Figure 9 | Effectiveness measure (Eff) components trends with Human Development Index (HDI).** For each individual country (listed in Extended Data Table 2), results are shown for: exponents **(a)** (β_d_ - β_s_), **(b)** β_s_ and **(c)** β_d_, and scale factors **(d)** (Ad/As), **(e)** As and **(f)** Ad (with index s and d indicating supply or demand, respectively). Purple and orange lines/symbols distinguish city-average ratio of realized to potential ES, and city-area fraction with high degree (≥0.5) of local ES metrics respectively, with blue and brown symbol outlines showing eastern and western countries respectively. Solid lines show best log/power law fits for all countries, with associated equation y and coefficient of determination R^2^ values also given in each panel.


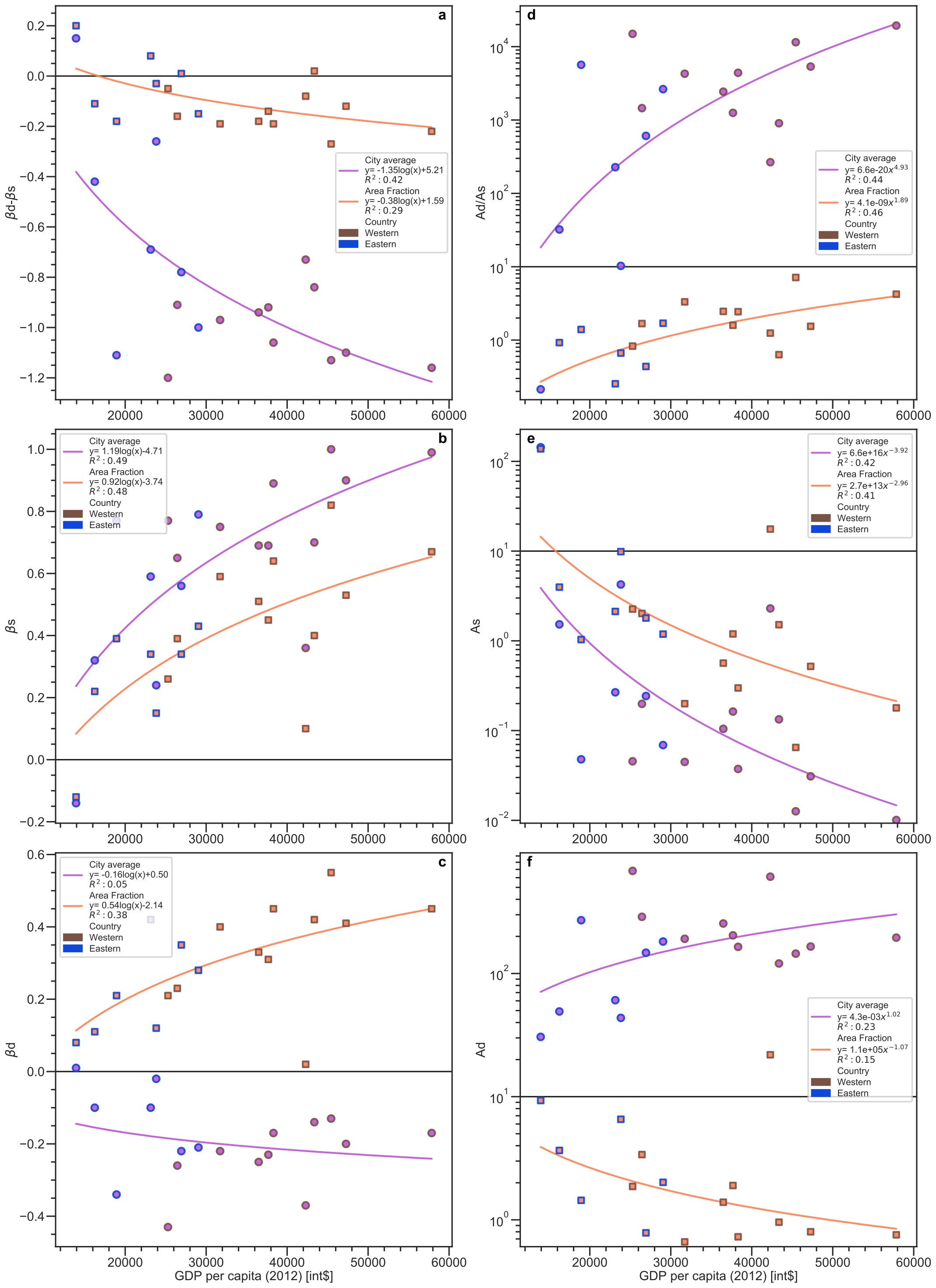


**Supplementary Figure 10 | Effectiveness measure (Eff) components trends with GDP per capita.** For each individual country (listed in Extended Data Table 2), results are shown for: exponents **(a)** (β_d_ - β_s_), **(b)** β_s_ and **(c)** β_d_, and scale factors **(d)** (Ad/As), **(e)** As and **(f)** Ad (with index s and d indicating supply or demand, respectively). Purple and orange lines/symbols distinguish city-average ratio of realized to potential ES, and city-area fraction with high degree (≥0.5) of local ES metrics respectively, with blue and brown symbol outlines showing eastern and western countries respectively. Solid lines show best log/power law fits for all countries, with associated equation y and coefficient of determination R^2^ values also given in each panel.

**Supplementary Note 1 | Quantification example for Stockholm (Supplementary Fig. 3), in the city boundary**. In bold we highlight the general metrics reported in the main article and Supplementary material.

**Stockholm city area** $\approx$ 1361 km^2^, **Population density** $\approx$ 1195 people/km^2^

| ${Ps}_{global}=\sum{Ps}_{local}=11852761$,  with Ps_local_ the individual per pixel Ps score | ${Pd}_{global}=\sum{Pd}_{local}=1936332$,  with Pd_local_ the individual per pixel Pd score |
| --- | --- |
| ${Rs}_{global}=\sum{Rs}_{local}=1112055$,  with Rs_local_ the individual per pixel Rs score | ${Rd}_{global}=\sum{Rd}_{local}=1441119$,  with Rd_local_ the individual per pixel Rd score |
| $\frac{{Rs}_{global}}{{Ps}_{global}}=\frac{1112055}{11852761}\times100\approx9\text{\%}$ | $\frac{{Rd}_{global}}{{Pd}_{global}}=\frac{1441119}{1936332}\times100\approx74\text{\%}$ |

| $Area\frac{{Rs}_{local}}{{Ps}_{local}}\approx251{km}^{2}$ , where $\frac{{Rs}_{local}}{{Ps}_{local}}\geq0.5$  In other words, the total area where the per pixel supply is at least half realized (consumed). | $Area\frac{{Rd}_{local}}{{Pd}_{local}}\approx327{km}^{2}$ , where $\frac{{Rd}_{local}}{{Pd}_{local}}\geq0.5$  In other words, the total area where the per pixel demand is at least half realized (fulfilled). |
| --- | --- |
| $\frac{Area\frac{{Rs}_{local}}{{Ps}_{local}}}{Areacity}\approx\frac{251}{1361}\times100\approx18\text{\%}$  In other words, the proportion of supply area at least half realized per city total area. | $\frac{Area\frac{{Rd}_{local}}{{Pd}_{local}}}{Areacity}\approx\frac{327}{1361}\times100\approx24\text{\%}$  In other words, the proportion of demand area at least half realized per city total area. |
